# Proteomic, Metabolomic, and Fatty Acid Profiling of Small Extracellular Vesicles from Glioblastoma Stem-Like Cells and Their Role in Tumor Heterogeneity

**DOI:** 10.1021/acsnano.3c11427

**Published:** 2024-01-11

**Authors:** Tolga Lokumcu, Murat Iskar, Martin Schneider, Dominic Helm, Glynis Klinke, Lisa Schlicker, Frederic Bethke, Gabriele Müller, Karsten Richter, Gernot Poschet, Emma Phillips, Violaine Goidts

**Affiliations:** † Brain Tumor Translational Targets, German Cancer Research Center (DKFZ), Heidelberg 69120, Germany; ‡ Faculty of Biosciences, University of Heidelberg, Heidelberg 69120, Germany; § Friedrich Miescher Institute for Biomedical Research, Basel 4058, Switzerland; ∥ Proteomics Core Facility, German Cancer Research Center (DKFZ), Heidelberg 69120, Germany; ⊥ Metabolomics Core Technology Platform, Centre for Organismal Studies, Heidelberg University, Heidelberg 69120, Germany; # Division of Tumor Metabolism and Microenvironment, German Cancer Research Center (DKFZ), Heidelberg 69120, Germany; ∇ Core Facility Electron Microscopy, German Cancer Research Center (DKFZ), Heidelberg 69120, Germany

**Keywords:** small extracellular vesicles, exosomes, metabolomics, proteomics, fatty acids, glioblastoma, glioblastoma stem-like cells

## Abstract

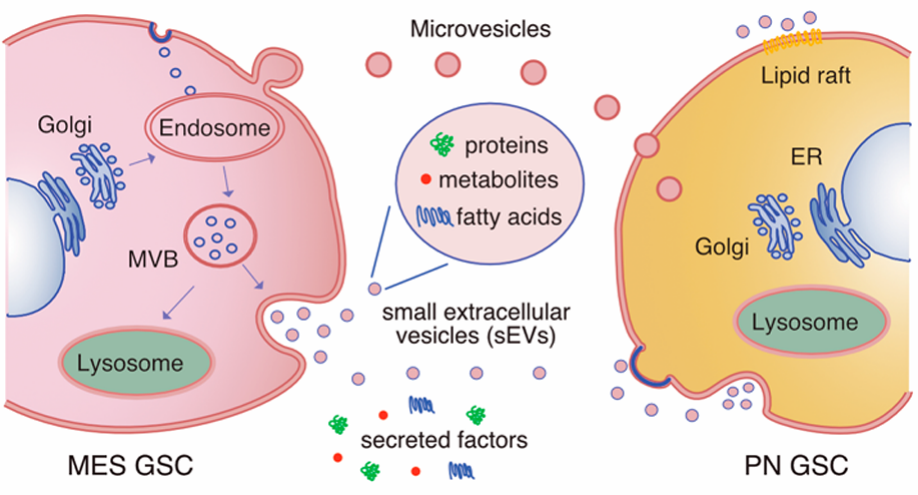

Glioblastoma is a deadly brain tumor for which there is no cure. The presence of glioblastoma stem-like cells (GSCs) contributes to the heterogeneous nature of the disease and makes developing effective therapies challenging. Glioblastoma cells have been shown to influence their environment by releasing biological nanostructures known as extracellular vesicles (EVs). Here, we investigated the role of GSC-derived nanosized EVs (<200 nm) in glioblastoma heterogeneity, plasticity, and aggressiveness, with a particular focus on their protein, metabolite, and fatty acid content. We showed that conditioned medium and small extracellular vesicles (sEVs) derived from cells of one glioblastoma subtype induced transcriptomic and proteomic changes in cells of another subtype. We found that GSC-derived sEVs are enriched in proteins playing a role in the transmembrane transport of amino acids, carboxylic acids, and organic acids, growth factor binding, and metabolites associated with amino acid, carboxylic acid, and sugar metabolism. This suggests a dual role of GSC-derived sEVs in supplying neighboring GSCs with valuable metabolites and proteins responsible for their transport. Moreover, GSC-derived sEVs were enriched in saturated fatty acids, while their respective cells were high in unsaturated fatty acids, supporting that the loading of biological cargos into sEVs is a highly regulated process and that GSC-derived sEVs could be sources of saturated fatty acids for the maintenance of glioblastoma cell metabolism. Interestingly, sEVs isolated from GSCs of the proneural and mesenchymal subtypes are enriched in specific sets of proteins, metabolites, and fatty acids, suggesting a molecular collaboration between transcriptionally different glioblastoma cells. In summary, this study revealed the complexity of GSC-derived sEVs and unveiled their potential contribution to tumor heterogeneity and critical cellular processes commonly deregulated in glioblastoma.

## Introduction

Glioblastoma (GBM) is the most frequent brain and central nervous system (CNS) tumor, accounting for 48.6% of primary malignant brain tumors and 14.5% of all primary brain and CNS tumors. Despite the aggressive treatments with surgical resection, irradiation, and chemotherapy, glioblastoma still has a dismal prognosis with the median survival less than 15 months after diagnosis.^1,2^

Glioblastoma is an extremely aggressive tumor showing a high degree of intra- and intertumoral heterogeneity, which creates a major challenge for implementing better treatment strategies. Gene expression and methylation profiling studies helped to define prognostic and subtype specific signatures, allowing the classification of high-grade gliomas (HGGs), including glioblastoma. Phillips and co-workers, using gene expression profiling, defined a gene signature to stratify HGGs into subclasses named proneural (PN), mesenchymal (MES), and proliferative, relating PN and MES to favorable and poor outcomes, respectively.^3^ In addition, subsequent analysis of The Cancer Genome Atlas (TCGA) expression profile data revealed four glioblastoma subtypes termed as proneural (PN), neural (NE), classical (CL), and mesenchymal (MES).^4^ After distinguishing glioblastoma-specific mRNAs from those associated with nontumor cells, glioblastoma subtypes were revised to proneural, classical, and mesenchymal.^5^ Moreover, Neftel et al., using single-cell RNA sequencing of glioblastoma specimens and bulk expression analysis of TCGA samples, elucidated the different cellular states and plasticity of these malignant cells, revealing high heterogeneity in glioblastoma.^6^

Previous studies revealed that glioblastoma cells have a striking ability to influence their environment through extracellular vesicles to facilitate their growth and invasive potential. For instance, glioblastoma cells stimulate tubule formation by delivering angiogenic proteins to surrounding endothelial cells via microvesicles, resulting in increased angiogenesis and malignancy.^7^ Glioblastoma cells have also been shown to exchange oncogenic proteins such as epidermal growth factor receptor variant III (EGFRvIII), which can confer a growth advantage and increased invasiveness.^8^

Emerging evidence suggests that altered tumor metabolism is a defining hallmark of glioblastoma and that metabolic reprogramming contributes to the plasticity, heterogeneity, and therapeutic resistance of glioblastoma cells.^9−11^ In addition to the dependence of cancer cells on aerobic glycolysis, recent studies have demonstrated that fatty acid metabolism also plays a crucial role in tumorigenesis. Fatty acid oxidation was recently shown to be one of the key drivers of progression from low-grade gliomas into high-grade glioblastomas.^12^ Another study demonstrated the presence of enzymes involved in fatty acid oxidation within human glioblastoma tissues and showed that the inhibition of fatty acid oxidation diminished tumor growth and prolonged survival *in vivo*.^13^ Furthermore, reduced expression of acyl-CoA-binding protein (ACBP), a protein that mediates fatty acid oxidation, led to tumor senescence and prolonged the survival of experimental animals, further highlighting the dependence of glioblastoma cells on fatty acid metabolism.^14^

It has been previously demonstrated that glioblastoma stem-like cells are highly plastic and can transition from one subtype to another one dynamically, which is a phenomenon known as proneural-to-mesenchymal transition (PMT) in glioblastoma.^3,15,16^ This epithelial-to-mesenchymal (EMT)-like shift of glioblastoma cells is accompanied by a higher resistance to therapy.^15,16^ However, the molecular mechanisms responsible for the transition from the proneural to the mesenchymal subtype are ill-defined.

In order to identify molecules that might be involved in this plasticity, we investigated the content of small extracellular vesicles (sEVs, approximately <200 nm diameter) from different glioblastoma subtypes. To assess the contribution of sEVs to the plastic and highly heterogeneous nature of glioblastoma cells, we profiled their protein, metabolite, and fatty acid content. We showed that GSC-derived sEVs harbor proteins, metabolites, and fatty acids, which are biologically critical for GSC cell metabolism, plasticity, aggressiveness, and heterogeneity. In addition, we revealed the differential abundance of certain biomolecules in PN and MES sEVs, suggesting cooperation between transcriptionally different GSC subtypes by means of sEVs, contributing to the aggressive nature of glioblastoma.

## Results

### GSC-Secreted Biomolecules Are Involved in Cancer Cell Plasticity and Tumor Heterogeneity

**Figure 1:**
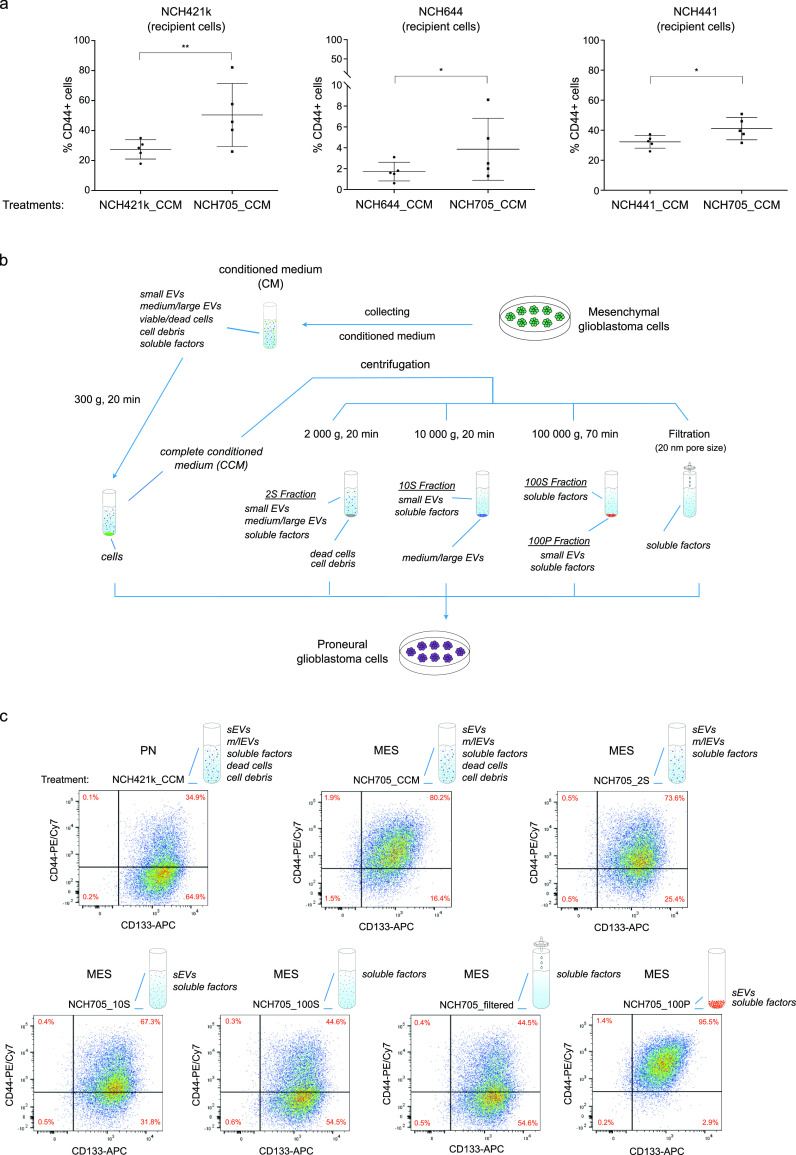
Treatment of proneural cells with mesenchymal conditioned medium. (a) PN cells (NCH421k, NCH644, and NCH441) were treated with the complete conditioned medium of mesenchymal NCH705 cells, and percent CD44-positivity was measured by flow cytometry. *p < 0.05, **p < 0.01, as determined by ratio paired *t* test. Mean with SD of *n* = 5 biological replicates is shown. (b) A scheme depicting the fractionation of complete conditioned medium by ultracentrifugation and filtration. CM, conditioned medium; CCM, complete conditioned medium; 2S, 10S, and 100S Fractions, supernatant obtained after centrifugation at 2000*g*, 10,000*g*, and 100,000*g*, respectively; 100P Fraction, pellet obtained from 100,000*g* centrifugation; Filtered, flow-through after filtration with 0.02 μm-membrane filter. (c) Flow cytometry results of PN cells treated with the different fractions of MES conditioned medium. NCH421k cells (PN) were treated with the different fractions of NCH705 (MES) conditioned medium, and the expression of PN and MES GSC surface markers (CD133 and CD44, respectively) was measured by flow cytometry. NCH421k complete conditioned medium (NCH421k_CCM) was used as a control.

To investigate the role of GSC-secreted biomolecules in glioblastoma heterogeneity and cell plasticity, proneural (NCH421k, NCH644, NCH441) and mesenchymal (NCH705, NCH711d) patient-derived glioblastoma stem-like cells were used, which were classified into subtypes with single-sample gene set enrichment analysis (ssGSEA)^17^ using the Wang gene signatures.^5^ In this regard, proneural cells were treated with the conditioned medium from mesenchymal cells, and the abundance of CD44, a well-characterized mesenchymal GSC marker, was measured by flow cytometry. Proneural cells treated with mesenchymal conditioned medium increased CD44 expression significantly (Figure 1a). Subsequently, to examine which biomolecules are responsible for the increase in CD44 expression, mesenchymal conditioned medium was fractionated by centrifugation and filtration, separating soluble factors and small and medium/large EVs (Figure 1b). Proneural cells treated with the mesenchymal supernatant obtained after centrifugation at 2000*g* and 10,000*g*, called 2S and 10S fractions, respectively, showed an increase in CD44 abundance by flow cytometry analysis, comparable with the PN cells treated with the MES complete conditioned medium (CCM) (Figure 1c and Supporting Information Figure S1a-c). Interestingly, this effect was abrogated when small extracellular vesicles (sEVs) were depleted from the supernatant by 100,000*g* centrifugation (100S) and filtering with a membrane filter (pore size, 20 nm), suggesting that sEVs may contain important factors that drive the increase in CD44 expression in recipient cells. Indeed, treating PN cells with the sEV-enriched fraction (100P) also resulted in the increase of CD44 abundance, which was not observed when they were exposed to the soluble factor-enriched fraction only (Filtered), further indicating that the sEVs might contain relevant factors for tumorigenesis.

**Figure 2:**
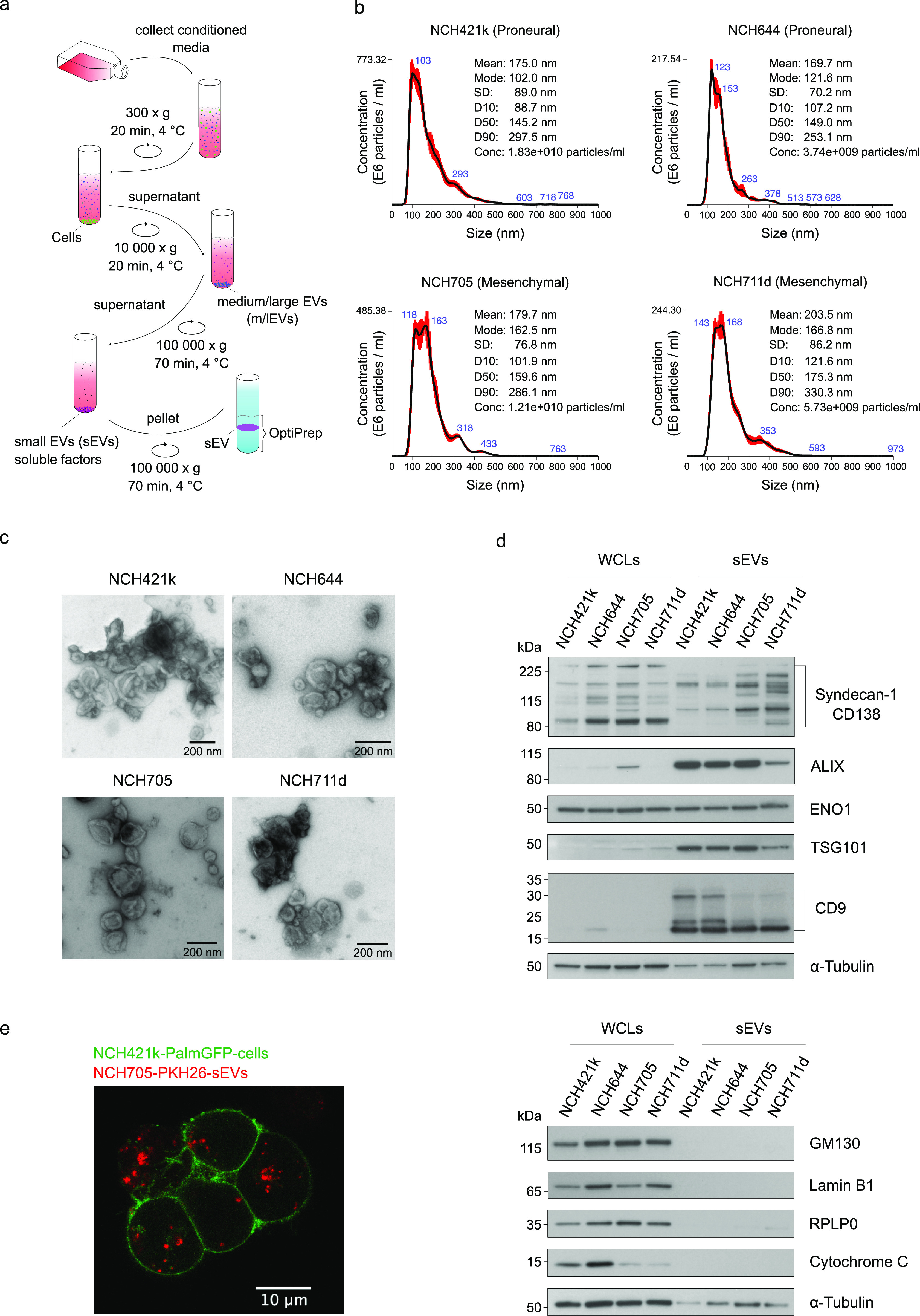
Separation, characterization, and internalization of sEVs. (a) Separation of sEVs using dUC followed by OptiPrep density cushion. (b) NTA of sEVs separated from NCH421k, NCH644, NCH705, and NCH711d cells. Data were obtained from five 60-s videos for each sample. The red error bars indicate ±1 standard error of the mean. The mean size, modal size, standard deviation (SD), percent undersize (D10, D50, and D90), and sample concentration are shown in upper right of the plots. The size distributions are represented as mean (black line) with standard error (red area). (c) Negative-stain TEM images (close-up) of NCH421k, NCH644, NCH705, and NCH711d sEVs. Scale bar, 200 nm. (d) Western blots of NCH421k, NCH644, NCH705, and NCH711d whole-cell lysates (WCLs) and sEVs. EV markers (CD138, ALIX, ENO1, TSG101, and CD9), Golgi marker (GM130), mitochondrial marker (Cytochrome C), nuclear protein (Lamin B1), and ribosomal protein (RPLP0) are depicted. α-Tubulin was used as a loading control for WCLs. (e) Representative confocal microscopy image visualizing the internalization of PKH26-labeled mesenchymal sEVs (NCH705-PKH26-sEVs) by PalmGFP expressing proneural NCH421k cells (NCH421k-PalmGFP-cells).

To thoroughly characterize the sEVs from different subtypes, GSC-derived sEVs were separated using differential ultracentrifugation (dUC) followed by an OptiPrep density cushion (Figure 2a), and their size distribution was assessed by nanoparticle tracking analysis (NTA). The separated sEVs had a size distribution of ∼170–200 nm (Figure 2b), as previously reported,^18,19^ and the transmission electron microscopy (TEM) micrographs revealed the size (≲200 nm) of EVs and visualized them with typical cup-shaped morphology after negative staining, confirming that the preparations were enriched in sEVs (Figure 2c and Supporting Information Figure S2a). Protein content-based characterization of sEVs by Western blotting confirmed the high expression of sEV markers CD138, ALIX, ENO1, TSG10, and CD9 (Figure 2d, top panel). To attribute the specificity of the study to sEVs, the depletion of markers (GM130, lamin B1, and cytochrome C), which are associated with other intracellular compartments than plasma membrane and endosome, was verified. In addition, the near absence of potential coisolate RPLP0 (a ribosomal protein) indicated the high degree of purity of sEV preparations (Figure 2d, bottom panel). To verify the uptake of sEVs by GSCs, fluorescently labeled PN cells (NCH421k-PalmGFP-Cells) were treated with PKH26-stained MES sEVs (NCH705-PKH26-sEV) or genetically labeled MES sEVs (NCH705-PalmtdTomato-sEVs), and the internalization of sEVs was monitored by confocal microscopy (Figure 2e and Supporting Information Figure S2b).

The uptake of sEVs was also determined over time via flow cytometry using PalmtdTomato tagged sEVs (NCH705-PalmtdTomato-sEVs), revealing the time-dependent internalization of sEVs by recipient cells (Supporting Information Figure S2c).

Taken together, these data show that patient-derived GSCs secrete sEVs and that sEVs derived from a subpopulation of GSCs can be internalized by a transcriptionally different subpopulation of GSCs and bring about a change in surface marker expression. This suggests that sEVs from GSCs may contribute to the high level of cell plasticity and heterogeneity observed in glioblastoma.

### GSC-Derived sEVs and Their Parental Cells Have Distinct Subtype-Specific Subsets of Proteins

**Figure 3:**
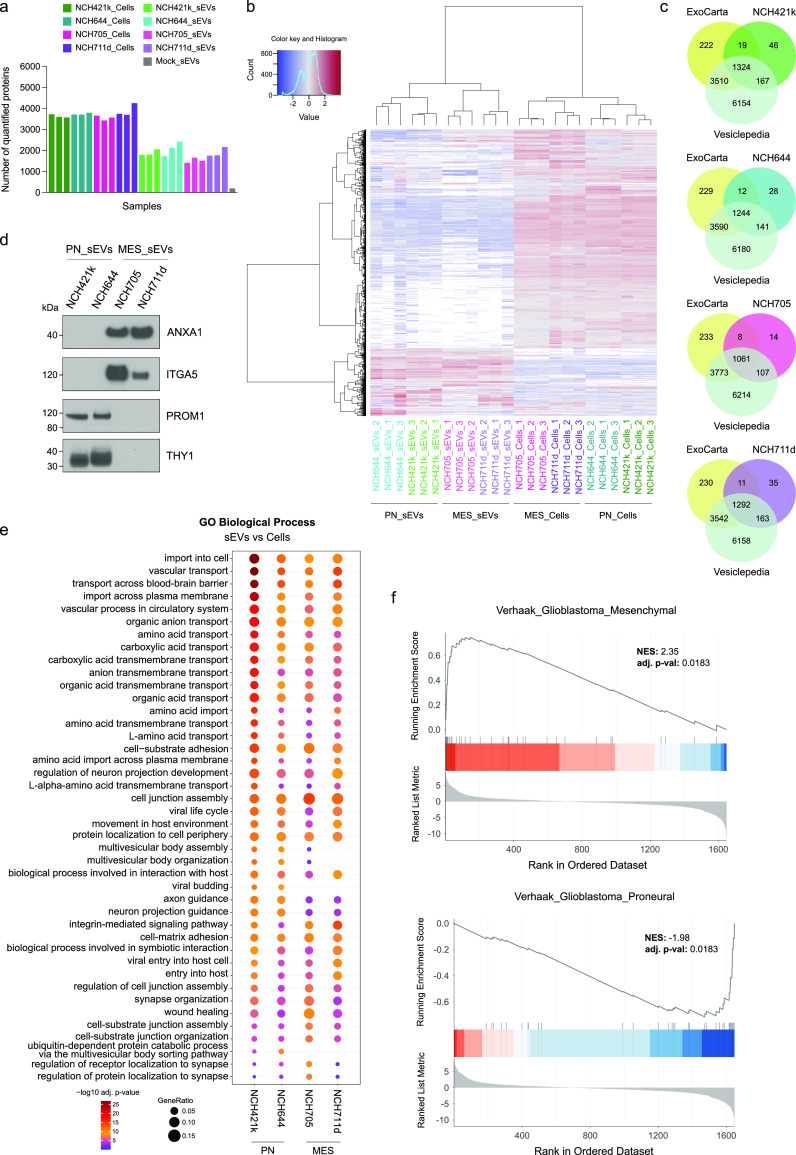
Mass spectrometry-based proteome profiling of GSCs and GSC-derived sEVs (*n* = 3 biologically independent samples). (a) Bar graph demonstrating the total number of proteins quantified in each sample. Mock_sEVs describes the sEV isolation from cell-free conditioned medium. (b) Hierarchical clustering heatmap of z-score normalized log_2_ LFQ intensities. Hierarchical clustering on rows and columns was generated with Euclidean distances and Ward.D2 clustering. (c) Venn diagrams showing the number of proteins identified in the sEVs samples (in all triplicates) compared with the proteins listed in the vesicle proteome data sets Vesiclepedia and ExoCarta. (d) Western blots of selected proteins (Annexin A1, Integrin alpha-5, Prominin-1, and Thy1) to validate MS-based proteome profiling results. (e) Gene ontology (GO) enrichment analysis of biological process for differentially abundant proteins (sEVs vs Cells, adj. pval <0.001 and log_2_FC > 4, biological triplicates) in sEVs. The plot was generated by combining the 20 most significantly enriched (ranked by adjusted p value, Benjamini-Hochberg) biological process terms of each sEVs vs cells comparisons. Dot size indicates the gene ratio. (f) Gene set enrichment analysis (GSEA) of proteins differentially expressed in MES sEVs compared to PN sEVs.

Next, the protein content of GSC-derived sEVs, as well as their respective parental cell lines, was determined by liquid chromatography–mass spectrometry (LC-MS/MS) in triplicate. The number of proteins quantifiable in each sample (i.e., label-free quantification (LFQ) value determined by the search engine) is displayed in Figure 3a.

To identify clusters of proteins and sEV/cell samples with consistent behavior, hierarchical clustering was performed using Euclidean distance and Ward’s D2 clustering. The relative LFQ intensities across the samples allowed us to visualize the subtype specific clusters of proteins in sEVs and their respective GSC lines (Figure 3b). Further, principal component analysis (PCA) demonstrated a clear cluster separation of the proneural and mesenchymal sEVs, which was also true for their parental cells (Supporting Information Figure S3a). In addition, all biological replicates of sEVs and cell samples cluster tightly together, highlighting the robustness of the sEV separation, sample preparation, and MS quantification, which is in line with the unsupervised hierarchical clustering of the Pearson correlations (Supporting Information Figure S3b). To verify whether sEV proteins identified in this study have also been previously associated with the extracellular vesicles, MS data was cross-referenced with the publicly available extracellular vesicles proteome databases Vesiclepedia and ExoCarta.^20,21^ Initially, proteins listed in these two databases were sorted according to the identification method (“mass spectrometry”) and species (“Homo sapiens”), and the sorted list was used as a reference to evaluate the degree of match with the glioblastoma sEVs. Venn diagrams demonstrate that the majority of GSC-derived sEV proteins identified in this study (in all triplicates) have been previously reported to be expressed in extracellular vesicles, further verifying the efficient sEV isolation and robust MS quantification. Importantly, several proteins which have not been reported by previous mass spectrometry-based proteomic analyses of EVs have also been identified in our GSC-derived sEVs (Figure 3c). A total of 727 proteins were shared by all GSC-derived sEVs (in all triplicates), Vesiclepedia, and ExoCarta. Furthermore, 9 proteins, namely, solute carrier family 48 member 1 (SLC48A1), uronyl 2-sulfotransferase (UST), ilvB acetolactate synthase-like protein (ILVBL), leucine-rich repeat containing 8 VRAC subunit B (LRRC8B), lipase maturation factor 2 (LMF2), fibroblast growth factor binding protein 3 (FGFBP3), Ecm29 proteasome adaptor and scaffold (ECM29), signal peptide peptidase like 2B (SPPL2B), and MAM domain-containing glycosylphosphatidylinositol anchor protein 2 (MDGA2) were identified in all triplicates of PN sEVs but not in the other data sets, while plexin domain-containing protein 1 (PLXDC1) was the only protein found only in all triplicates of MES sEVs (Supporting Information Figure S3c).

Next, the differentially abundant proteins (adjusted p-value <0.001 and log_2_FC > 4) in sEVs compared with their respective cell lines were examined. Mesenchymal sEVs were found to be highly enriched in annexins (ANXA1, ANXA2), integrins (ITGA3, ITGA5, ITGB4), neuropilin-1 (NRP1), DBH-like monooxygenase protein 1 (MOXD1), endoglin (ENG), cluster of differentiation 109 (CD109), matrix metalloproteinase-14 (MMP14), spectrin beta chain, nonerythrocytic 1 (SPTBN1), and trophoblast glycoprotein (TPBG) in comparison to proneural sEVs. On the other hand, proneural sEVs were high in adhesion G-protein coupled receptor G1 (GPR56), prominin-1 (PROM1), Thy-1 membrane protein (THY1), agrin (AGRN), contactin-1 (CNTN1), protein tweety homologue 1 (TTYH1), tetraspanin-7 (TSPAN7), and chondroitin sulfate proteoglycan 4 (CSPG4), further suggesting that the proteome of glioblastoma sEVs differ from subtype to subtype, which contributes to the heterogeneous nature of the disease (Supporting Information Figure S4). Furthermore, we validated our mass-spectrometry results by Western blotting of selected proteins, showing the enrichment of Annexin A1 (ANXA1) and Integrin alpha-5 (ITGA5) in MES sEVs, and Prominin-1 (PROM1) and Thy-1 membrane protein (THY1) in PN sEVs (Figure 3d).

### GSC-Derived sEVs Are Enriched in Proteins Related to Metabolic- and Cancer-Associated Pathways and Retain Their Subtype Characteristics

To gain functional insight into the proteome of sEVs, the relative abundance of proteins in sEVs and their respective cell line was compared. Gene ontology (GO) analysis of biological processes for differentially abundant proteins in sEVs showed significant enrichment of biological programs associated with the transmembrane transport, cell-matrix adhesion, and cell junction organization, in line with the well-recognized roles of sEVs in these processes. Interestingly, many other biological processes involving amino acid, carboxylic acid, and organic acid transmembrane transport, amino acid import, and organic anion transport were identified to be enriched in each sEV sample in comparison to their respective whole cell lysates, suggesting the involvement of tumor-secreted sEVs in metabolic pathways and fatty acid metabolism. In addition, the enrichment of proteins related to cancer-associated pathways and processes, such as integrin-mediated signaling, insulin-like growth factor (IGF), transforming growth factor beta (TGFβ), integrin, and cytokine binding, also implies the potential contribution of GSC-derived sEVs to the aggressive nature of glioblastoma (Figure 3e and Supporting Information Figure S5a). Moreover, GO analysis of cellular components for the proteins enriched in sEVs exhibited their association with plasma membrane, cell surface, adhesion, and endosome-related cellular component terms, which is in line with the biogenesis and mechanisms of secretion of sEVs (Supporting Information Figure S5b). Interestingly, sEVs produced from GSCs retained their subtype characteristics as shown by gene set enrichment analysis (GSEA) using the Verhaak glioblastoma subtype signatures^4^ (Figure 3f).

In order to contextualize our proteasome profiling results, we compared our data to previous studies.^22,23^ The study carried out by Ricklefs et al. using other patient-derived glioblastoma cell lines from the PN and MES subtypes assigned fewer proteins in total in their sEVs to be subtype-specific (14 PN and 75 MES). The upset plot (Supporting Information Figure S6) demonstrates that while we provide 326 and 230 additional proteins found in MES and PN sEVs, respectively, we also have some proteins in common, such as CD44 in MES sEVs or lipoprotein lipase (LPL) in PN sEVs. However, some of the proteins we identified in MES sEVs were identified in Ricklefs et al.’s PN sEVs and vice versa, and some of the proteins they assigned as specific to a particular subtype were not significantly different between our PN and MES sEVs. Additionally, Spinelli and co-workers demonstrated that MES sEVs were high in some of the EV-related tetraspanins such as CD9, CD63, and CD81 in comparison to PN sEVs; however, we identified that CD9 was significantly high in PN sEVs and that neither CD63 nor CD81 were differentially abundant in either subtype. In order to compare our data more broadly to the data presented by Ricklefs et al. and Spinelli et al., we also performed a GO analysis of our PN versus MES sEVs (Supporting Information figure S7). Similar to these studies, we also found that PN sEVs were enriched in proteins playing roles in nervous system development (axon development, axonogenesis, gliogenesis), structural integrity of ECM (extracellular matrix structural constituent), cell communication (cell junction assembly), signal transduction pathways (growth factor receptor binding, integrin binding), and proteins associated with plasma membrane and lysosome. Interestingly, our GO analysis additionally revealed PN sEVs to be enriched in proteins associated with cytoplasmic vesicle (endocytic vesicle, coated vesicle, clathrin-coated vesicle, clathrin-coated endocytic vesicle), endomembrane system (early endosome, recycling endosome, clathrin-coated pit, clathrin coat of coated pit), and transmembrane transporter (carboxylic acid/organic acid transmembrane transporter activity), whereas our MES sEVs are enriched in proteins regulating critical cellular events such as actin filament-based processes, cell junction assembly, cell adhesion/migration, cell projection organization, enzyme/protein/phospholipid binding, and lipase inhibitor activity.

### The CM and sEVs Derived from One Subtype Induced Transcriptomic and Proteomic Changes of Another Subtype

**Figure 4:**
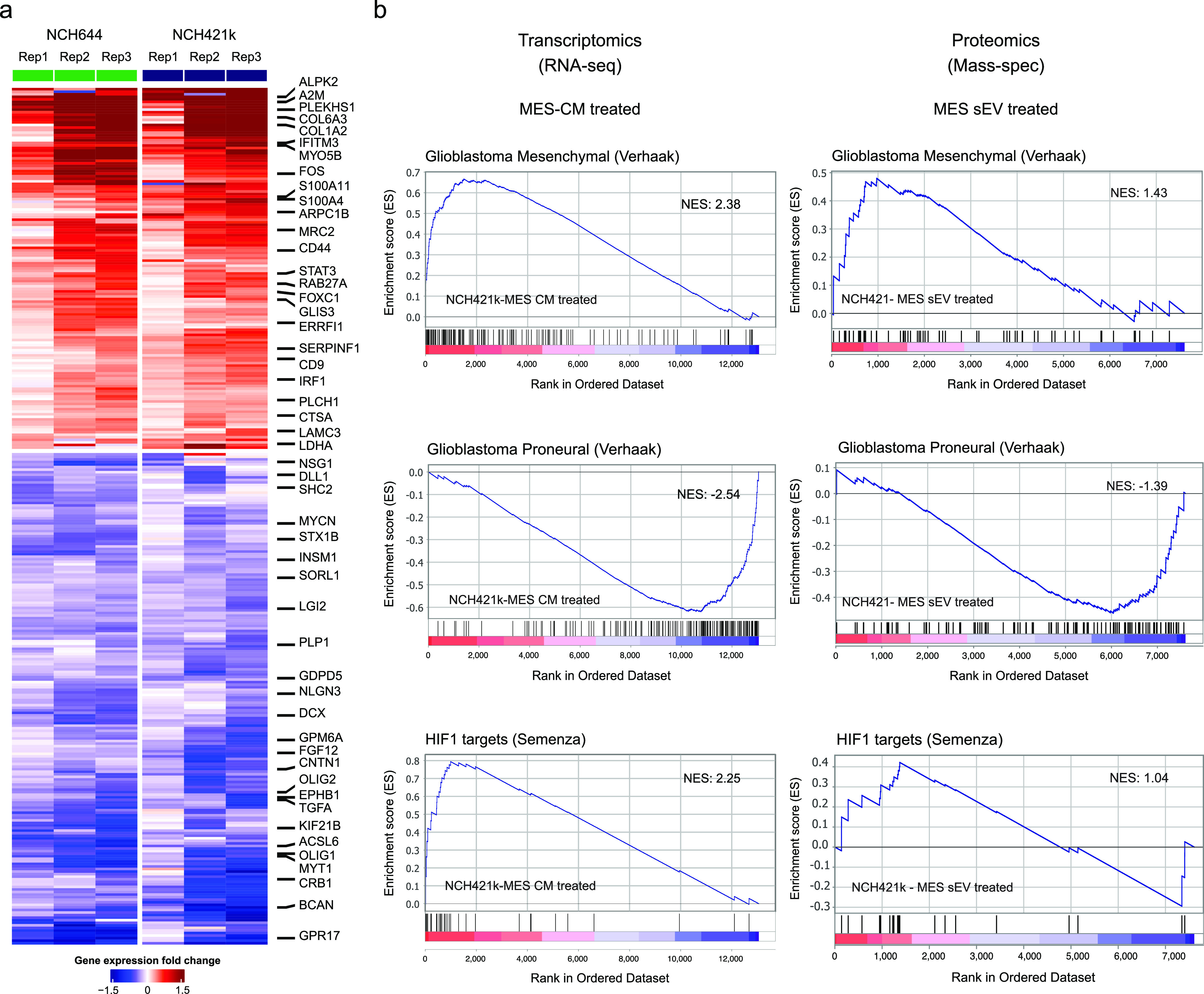
Transcriptomic and proteomic analysis of PN cells treated with the CM/sEVs of MES cells. (a) Heatmap depicting the RNA-sequencing results of PN GSCs (NCH421k and NCH644) treated with MES-CM (NCH705) or their own conditioned medium (control). The heatmap shows significantly upregulated (adj. pval <0.05 and log_2_FC > 0.25) and downregulated (adj. pval <0.05 and log_2_FC ← 0.25) genes in PN cells upon MES-CM treatment. For simplicity, only 25 genes from each group have been annotated. (b) GSEA of RNA-sequencing (left) and mass-spectrometry (right) data of PN NCH421k cells treated with NCH705 MES CM and sEV, respectively.

To investigate the impact of MES conditioned medium (CM) and sEVs on recipient PN cells, we also treated NCH421k and NCH644 cells with NCH705-derived CM and/or sEVs and performed transcriptomic and proteomic analyses. The RNA-seq-based gene expression analysis of PN cells revealed several genes that were differentially expressed upon MES-CM treatment (Figure 4a and Supporting Information Figure S8a). In addition, the GSEA of RNA-sequencing and mass-spectrometry data demonstrated that NCH421 cells treated with MES CM and sEVs increased the expression of genes associated with the mesenchymal subtype of glioblastoma, while proneural subtype-related genes were downregulated. This also held true when NCH644 cells were treated with MES CM (Figure 4b and Supporting Information Figure S8b). In addition, both NCH421k and NCH644 cells demonstrated increased abundance of proteins regulated by hypoxia-inducible factor HIF1 activity upon MES CM and sEV treatment, which was in line with the RNA-sequencing of MES CM treated PN cells.

### Untargeted Metabolite Profiling Revealed the Presence of Biologically Important Metabolites in GSC-Derived sEVs

Like most cancer cells, glioblastoma cells can also rewire their cellular metabolism to sustain their survival, growth, proliferation, invasion, and therapeutic resistance. Accordingly, we studied the potential role of glioblastoma-derived sEVs in rewiring cellular metabolism to support cellular plasticity and heterogeneity, which could potentially result in increased aggressiveness and resistance to conventional therapies.

**Figure 5:**
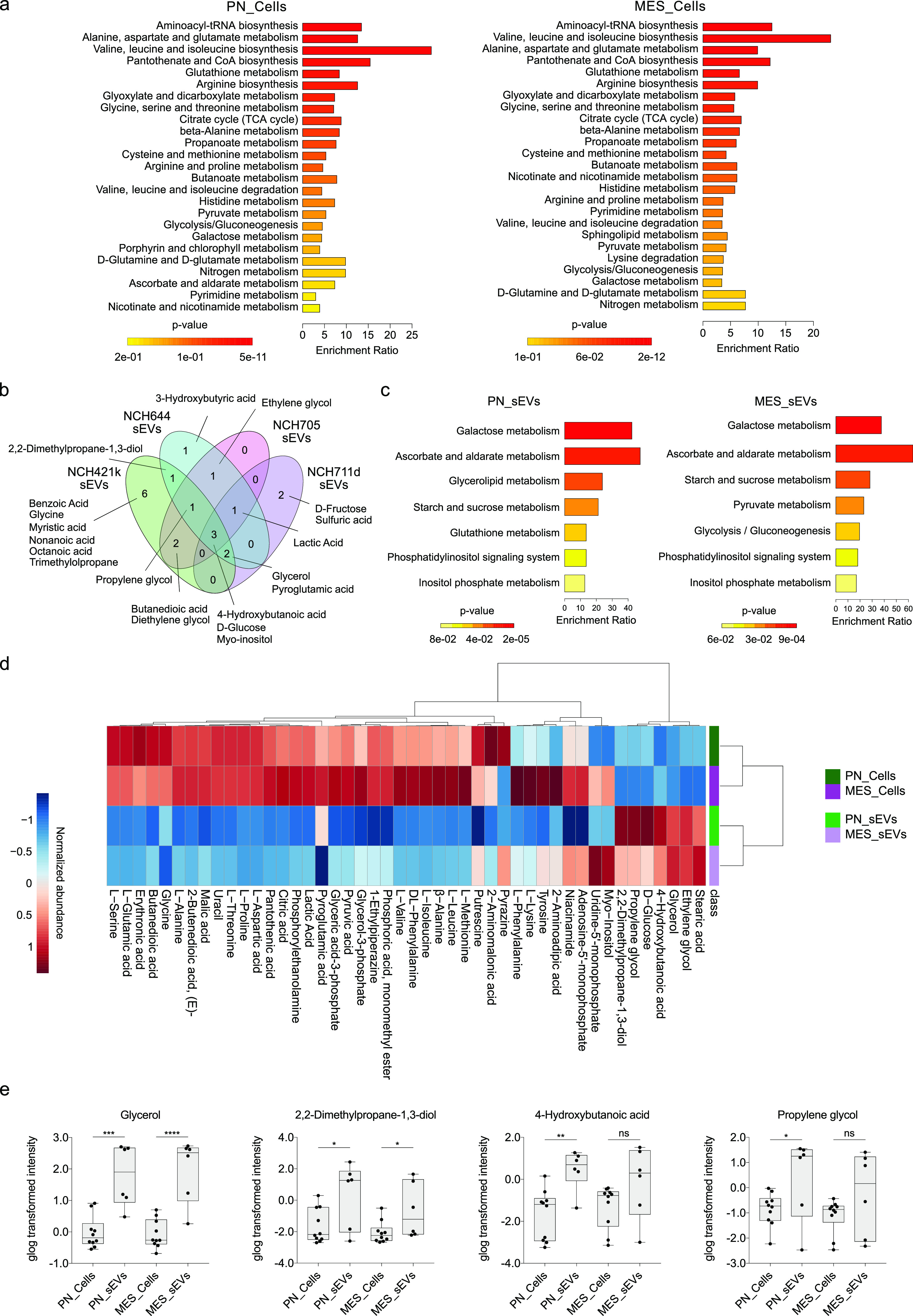
Untargeted metabolomic profiling of GSCs and GSC-derived sEVs. (a) MSEA displaying the enrichment of metabolic sets in PN (left) and MES GSCs (right). Metabolite sets enriched in PN and MES cells were determined by over representation analysis (ORA) using the hypergeometric test (MetaboAnalyst 5.0). KEGG human metabolic pathways containing at least 5 entries were used as a metabolite set library. The metabolite sets are ranked according to the p-value, and color intensity yellow to red indicates increasing statistical significance. The top 25 metabolite sets were demonstrated in the bar charts. The enrichment ratio represents the ratio of observed hits to expected hits. (b) Venn diagram showing the metabolites identified in proneural (NCH421k and NCH644) and mesenchymal (NCH705 and NCH711d) sEV samples. Only metabolites detected in at least two out of three replicates of a given sample were shown in the Venn diagram. (c) MSEA showing the enrichment of metabolite sets in PN (left) and MES (right) sEVs. Metabolites detected in at least two out of the three replicates for both sEV samples of a given subtype were used in the analysis. (d) The hierarchical clustering heatmap of metabolites identified in PN/MES cells and their sEVs. Distance measure: Euclidean, Clustering algorithm: Ward. The metabolomics data were normalized by sum. The heatmap shows the class averages. Green: proneural cells (PN_Cells); purple: mesenchymal cells (MES_Cells); light green: proneural small extracellular vesicles (PN_sEVs); light purple: mesenchymal small extracellular vesicles (MES_sEVs). Metabolites detected in at least three out of the five replicates for both parental cell lines of a given subtype and/or in at least two out of the three replicates for both sEV samples of a given subtype are indicated in the heatmap. (e) Box plots showing the statistically significant increase of glycerol, 2,2-dimethylpropane-1,3-diol, 4-hydroxybutanoic acid, and propylene glycol in PN and/or MES sEVs compared to their parental cells. The sum-normalized intensities were generalized logarithm (glog) transformed and statistically compared by unpaired *t* test (two-tailed). The significance was indicated as follows: * *p* < 0.05, ** *p* < 0.01, *** *p* < 0.001, **** *p* < 0.0001, ns: not significant. For simplicity, trimethylsilyl (TMS) derivatives were omitted from metabolite names indicated in Venn diagram (b), heatmap (d), and box plots (e). For full names, please refer to Supporting Information file 2.

Gas chromatography–mass spectrometry (GC-MS)-based untargeted metabolite screening of sEVs and their parent cells revealed several metabolites that are implicated in essential metabolic pathways, suggesting their crucial role in cell viability and metabolic reprogramming of glioblastoma cells. To explore biologically meaningful metabolic patterns which were significantly enriched in PN and MES GSCs, we first performed metabolite set enrichment analysis (MSEA)^24^ using the metabolites detected in PN (36 metabolites) and MES (43 metabolites) GSCs (Figure 5a and Supporting Information Figure S9a). Accordingly, using 84 metabolic sets (based on Kyoto Encyclopedia of Genes and Genomes (KEGG) human metabolic pathways) as a metabolite set library, we found that metabolites associated with amino acid, carbohydrate, cofactor and vitamin metabolism, and with translation, were significantly enriched in both PN and MES GSCs. Subsequently, we identified that PN and MES sEVs commonly contain 4-hydroxybutanoic acid, d-glucose, and myo-inositol. In addition, both NCH421k and NCH644 PN sEVs were shown to harbor 2,2-dimethylpropane-1,3-diol, propylene glycol, glycerol, and pyroglutamic acid, while lactic acid was commonly detected in both NCH705 and NCH711d MES sEVs (Figure 5b). MSEA of metabolites detected in GSC-derived sEVs indicated that both PN and MES sEVs contain metabolites related to galactose, ascorbate-aldarate, and starch-sucrose metabolism. In addition, glycerolipid and pyruvate metabolism-related metabolites were also identified in PN and MES sEVs (p-value <0.05), respectively (Figure 5c and Supporting Information Figure S9b). Moreover, the hierarchical clustering heatmap of metabolites also demonstrated that PN and MES cells harbor a similar set of metabolites and the abundance of these metabolites slightly differs from subtype to subtype (Figure 5d). However, we identified that some amino acids, namely, l-methionine, l-leucine, l-isoleucine, and l-valine, were significantly high in MES GSCs in comparison to PN GSCs (Supporting Information Figure S9c), whereas glycine was enriched in PN GSCs. PN GSCs were also significantly enriched in erythronic acid. Furthermore, our metabolite screening demonstrated that most l-amino acids were enriched in both PN and MES GSCs but not in their corresponding sEVs. Interestingly, by comparing the abundance of metabolites detected in PN/MES sEVs with their parental cells, we determined that both glycerol and 2,2-dimethylpropane-1,3-diol were significantly enriched in PN and MES sEVs compared with their respective parental cells. In contrast to MES sEVs, PN sEVs were also found to be significantly enriched in 4-hydroxybutanoic acid and propylene glycol in comparison to their parental cells (Figure 5e).

### GSC-Derived sEVs Are Enriched for Saturated Free Fatty Acids (FFAs) and Cholesterol

In the 1920s, Otto Warburg and his colleagues discovered that tumors have a high rate of glucose consumption compared to most nontransformed tissues and that the majority of glucose consumed by tumor cells is fermented to produce lactate even in the presence of oxygen.^25,26^ After these observations, it has long been thought that cancer cells primarily use glucose for energy production. However, cancer cells also metabolize many other substances, including fatty acids, for their cellular maintenance.^27^

**Figure 6:**
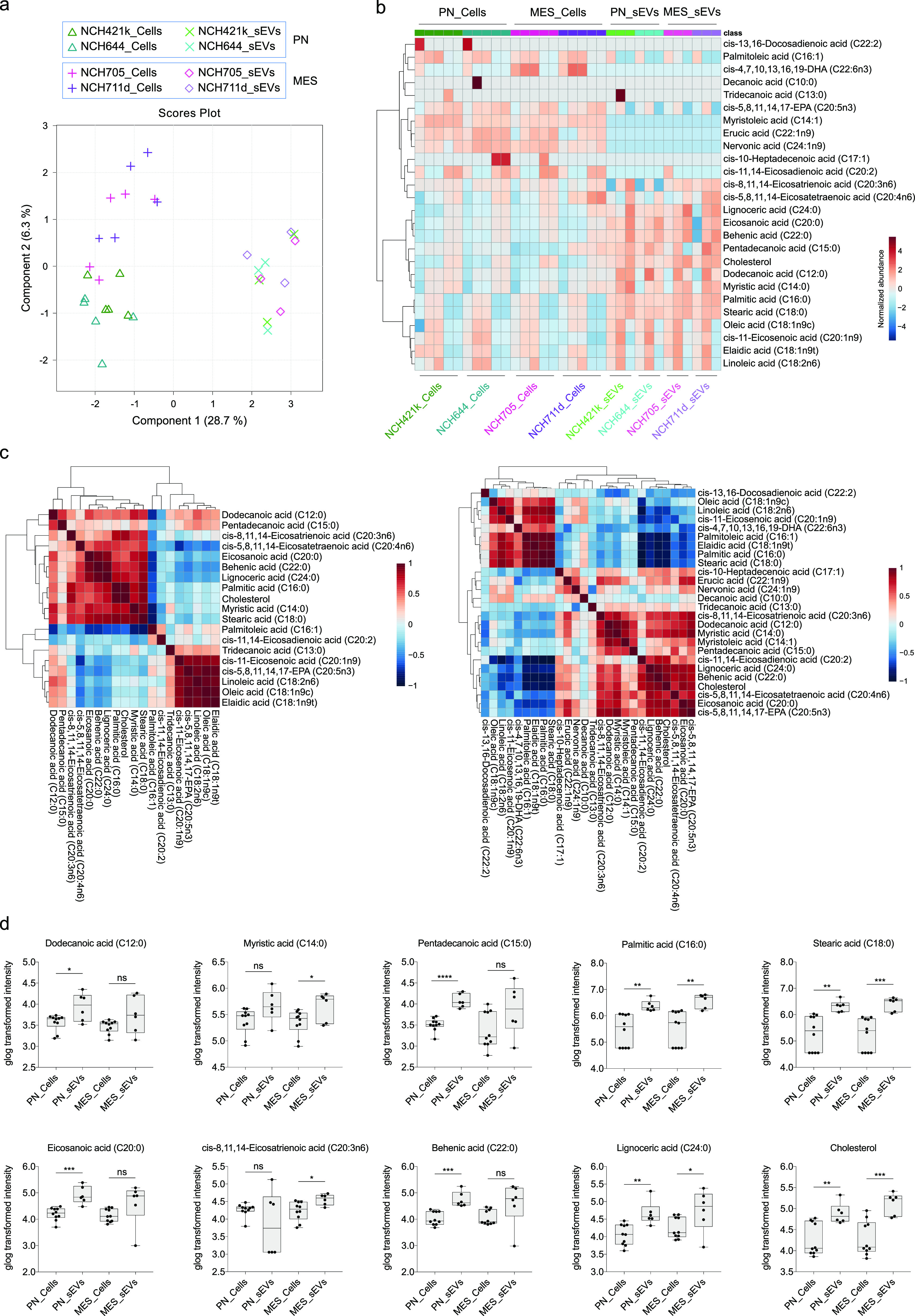
Fatty acid profiling of GSCs and GSC-derived sEVs. (a) 2-D scores plot using sPLS-DA of fatty acids. The symbols indicate biological replicates for GSC and sEV samples. (b) Hierarchical clustering heatmap of fatty acids in GSCs and sEVs. Distance measure: Euclidean, Clustering algorithm: Ward. (c) The correlation heatmap of FFAs detected in sEVs (left) and GSCs (right). Pearson correlation coefficient (Pearson’s *r*) was used as distance measure. Colors represent the Pearson’s coefficient of correlation: Red = positive, blue = negative correlation. (d) Box plots showing the statistically significant increase of dodecanoic acid (C12:0), myristic acid (C14:0), pentadecanoic acid (C15:0), palmitic acid (C16:0), stearic acid (C18:0), eicosanoic acid (C20:0), *cis*-8,11,14-eicosatrienoic acid (C20:3n6), behenic acid (C22:0), lignoceric acid (C24:0), and cholesterol in PN and/or MES sEVs compared to their parental cells. The normalized intensities of fatty acids were generalized logarithm (glog) transformed and statistically compared by unpaired *t* test (two-tailed). The significance was indicated as follows: * *p* < 0.05, ** *p* < 0.01, *** *p* < 0.001, **** *p* < 0.0001, ns: not significant.

Considering that extracellular vesicles are reservoirs of fatty acids and serve as a transporter for them,^28−30^ we also profiled the free fatty acids (FFAs) and cholesterol in GSC-derived sEVs and their respective parental cells using GC-MS to better understand their role in glioblastoma heterogeneity. In two-dimensional (2-D) scores plot of the results, sEV samples and their respective cell line samples cluster separately from each other along component 1, indicating that FFA content differs between the cells and the sEVs. There is also separation of the proneural (NCH421k and NCH644) and mesenchymal (NCH705 and NCH711d) cell clusters along component 2. On the other hand, there is no separation between proneural and mesenchymal sEVs (Figure 6a). Interestingly, a hierarchical clustering heatmap revealed that cholesterol and many of the saturated fatty acids were specifically enriched in sEVs compared with their respective cell lines, while unsaturated fatty acids were high in the source cells, further indicating the difference in the fatty acid composition of sEVs and their parent cells (Figure 6b). Furthermore, a correlation analysis of FFAs detected in sEVs revealed a high positive correlation among the saturated fatty acids (Figure 6c, left). Respectively, a positive correlation of FFAs detected in parental cells was observed among the majority of unsaturated fatty acids (Figure 6c, right). To identify the FFAs that are associated with the sEVs/cells in a subtype-dependent manner, the abundance of fatty acids in sEVs and cells were compared, and it was found that both PN and MES sEVs were high in palmitic acid (C16:0), stearic acid (C18:0), and lignoceric acid (C24:0) as compared to their respective parental cells. In addition, whereas PN sEVs were found to be significantly enriched in dodecanoic acid (C12:0), pentadecanoic acid (C15:0), eicosanoic acid (C20:0), and behenic acid (C22:0), MES sEVs were significantly enriched in myristic acid (C14:0) and *cis*-8,11,14-eicosatrienoic acid (C20:3n6) in comparison to their respective parental cells (Figure 6d). Furthermore, we also identified that both PN and MES sEVs were enriched in cholesterol as compared with their parental cells. On the other hand, unsaturated FFAs, namely, myristoleic (C14:1), palmitoleic (C16:1), *cis*-11,14-eicosadienoic (C20:2), *cis*-5,8,11,14,17-eicosapentaenoic (C20:5n3), erucic (C22:1n9), *cis*-4,7,10,13,16,19-docosahexaenoic (C22:6n3), and nervonic (C24:1n9) acids, were not detected or detected in very low abundance in PN and/or MES sEVs in comparison to their source GSC lines (Supporting Information Figure S10).

Finally, to estimate the discriminatory power of each individual fatty acid, a variable importance in projection (VIP) analysis was performed, and myristoleic (C14:1), palmitic (C16:0), palmitoleic (C16:1), stearic (C18:0), erucic (C22:1n9), and nervonic (C24:1n9) acids were determined to have a VIP score above 1 for all five components, indicating their importance for the group separation (Supporting Information Figure S11).

## Discussion

Glioblastoma is the most common and lethal primary brain tumor of the central nervous system, which accounts for approximately half of primary malignant brain tumors. Despite intensive treatment modalities, the eradication of glioblastoma is still extremely challenging.^1,2^ Many intrinsic and extrinsic cellular factors, such as genomic instability, changes in gene expression, clonal evolution of tumor cells, epigenetic deregulation, and complex interactions between the tumor cells and neighboring cells within the tumor stroma, contribute to the aggressive nature of glioblastoma.^31−36^ Past studies have focused on the contribution of the molecular and cellular outcomes of these factors on glioblastoma progression. However, the role of GSC-derived sEVs in glioblastoma heterogeneity, plasticity, and aggressiveness has been very sparsely investigated. In this study, we demonstrated that GSCs can internalize sEVs produced by a transcriptionally different subpopulation of GSCs, resulting in a phenotypic change that may contribute to the plastic and heterogeneous nature of tumor cells. To this end, our proteomic, metabolomic, and fatty acid profiling of GSC-derived sEVs and their parental cells revealed that PN and MES sEVs harbor a distinct set of proteins, metabolites, and fatty acids, which GSCs potentially take up to maintain the heterogeneous and aggressive nature of the tumor.

Over the last decades, tumor-derived sEVs have emerged as critical regulators of cell communication between cancer and the surrounding cells, and several studies have focused on their biological and functional role in cancer initiation, progression, metastasis, and therapy resistance.^37^ Proteomic studies in EVs have elucidated the roles of tumor-derived sEVs in different cancer entities including glioblastoma;^22,23,38^ however, studies on the role of sEVs derived from transcriptionally different subpopulations of GSCs in increased heterogeneity, plasticity, and aggressiveness of glioblastoma are limited. Comparing our results to previous studies, we found some proteins and associated GO processes in common in PN and MES derived-sEVs but also some differences. We also present many proteins to be associated with sEVs of a particular subtype which have not been previously reported, elaborating on the proteomic landscape of PN and MES sEVs. While the commonalities speak for the validity of our data, the differences are not altogether surprising in the face of the extreme intertumoral heterogeneity of glioblastoma and differences in mass spectrometer sensitivity and analysis thresholds. Here, we also demonstrated that GSC-derived sEVs are enriched in proteins related to transmembrane transport of amino acids, carboxylic acids, and organic acids, making sEVs sources of critical mediators required for the transfer of essential biomolecules associated with amino acid and fatty acid metabolism, which have previously been shown to be altered in glioblastoma.^12,39−43^ Further, the enrichment of proteins that facilitate the binding of growth factors to their receptors in sEVs also suggest that GSC-derived sEVs could contribute to the pathogenesis and progression of glioblastoma by facilitating the initiation of key signaling pathways commonly deregulated in malignant gliomas. Moreover, we demonstrated that GSC-derived sEVs reflected the molecular subtype of their parental cells, which supports the possibility of using sEVs separated from patient-derived GSCs as a potential biomarker source for subtyping,^44^ ultimately supporting the development of better treatment strategies against glioblastoma. Furthermore, our findings that CM and sEVs derived from MES GSCs induced transcriptomic and proteomic changes in recipient PN GSCs support the hypothesis that GSC-derived sEVs contribute to the plastic and heterogeneous nature of glioblastoma stem-like cells. Additionally, transcriptional and translational activation of genes, such as HIF1 target genes, that are known to drive glioblastoma progression, further suggests that GSC-derived sEVs contribute to the aggressiveness of glioblastoma.

Altered tumor metabolism is a hallmark of glioblastoma. Metabolic reprogramming is critical for glioblastoma cells to maintain their cellular energetics, plasticity, and therapy resistance.^9−11,45,46^ In this study, we screened the metabolites of GSC-derived sEVs and their source cells to get an insight into their complexity and potential contribution to deregulated glioblastoma metabolism. We identified that both PN and MES sEVs were enriched in glycerol. In addition to its primary role as a structural component of the major classes of lipids,^47^ glycerol is also a very important intermediate in carbohydrate and lipid metabolism. The enzyme glycerol kinase converts glycerol to glycerol 3-phosphate (G3P), which is then oxidized to dihydroxyacetone phosphate (DHAP) by glycerol-3-phosphate dehydrogenase (GPD). Subsequently, the glycolytic intermediate DHAP is converted to glyceraldehyde 3-phosphate, which either undergoes gluconeogenesis to generate glucose or is oxidized via glycolysis.^48^ Intriguingly, a previous study revealed that dormant glioma stem cells, but not neural stem cells, highly express glycerol 3-phosphate dehydrogenase 1 (GPD1) *in vivo* and that the inhibition of GPD1 impairs glioblastoma stem cell maintenance pathways, resulting in prolonged survival in mouse models of glioblastoma.^49^ In addition, GPD1 loss has also been shown to downregulate the key regulators of the mTOR (mechanistic target of rapamycin) pathway, which is known to modulate glucose,^50,51^ amino acid,^52^ nucleotide,^53,54^ fatty acid, and lipid metabolism.^55−58^ Given also that G3P is a substrate for acyltransferases to generate phosphatidic acid (PA), a phospholipid required for the stability and activity of mTOR complexes,^59−61^ tight regulation of glycerol metabolism is critical for the cellular metabolism and dormancy of glioblastoma stem cells. We therefore speculate that sEV-mediated transfer of glycerol between GSCs is a supportive process that GSCs might use to preserve their cellular characteristics and maintain their adaptation to the challenging glioblastoma microenvironment. Additionally, we also identified that GSC-derived sEVs harbor 2,2-dimethylpropane-1,3-diol; however, biological and functional reasons behind its preferential loading to sEVs remains elusive and needs to be addressed in future studies.

In addition to glycerol, our metabolite screening also revealed that PN sEVs are significantly enriched in propylene glycol (1,2-propanediol), which arises during the detoxification of deleterious glycolysis byproducts.^62−65^ Specifically, the highly glycating agent methylglyoxal (MG) is produced through fragmentation of the glycolytic intermediates glyceraldehyde-3-phosphate (GA3P) and DHAP,^63^ spontaneously reacting with proteins, lipids, and nucleic acids, forming advanced glycation end products (AGEs).^64,66,67^ MG can be detoxified in several ways, including reduction by aldo-keto reductases (AKRs) to d-lactaldehyde and acetol, the latter of which is further metabolized to propylene glycol.^65^ Therefore, the enrichment of propylene glycol in PN sEVs might suggest the presence of an additional mechanism in GSCs for its excretion by means of sEVs, limiting the deleterious accumulation of methylglyoxal and its side-products. Supportively, Oizel and co-workers indicated that primary glioblastoma cells cluster into two metabolic phenotypes, glutamine^high^ (GLN^high^) and glutamine^low^ (GLN^low^), based on their glutamine consumption, and that GLN^high^ cells exhibit a mesenchymal signature, while GLN^low^ cells mostly belong to proneural or proliferative subclasses.^68^ Furthermore, they revealed that compared to GLN^high^ cells, GLN^low^ cells relied more on glycolysis, which might explain why PN sEVs but not MES sEVs significantly enriched in propylene glycol. However, the rate of glycolysis and consequent accumulation of deleterious byproducts in PN and MES GSCs should be further investigated to fully understand the reasons behind preferential loading of propylene glycol into PN sEVs.

Alterations in metabolism could drive phenotypic differentiation of GSCs. As such, metabolic deregulation of gamma-aminobutyric acid (GABA) catabolism in GSCs has been displayed to increase the level of its byproduct 4-hydroxybutanoic acid (gamma-hydroxybutyrate, GHB), promoting a switch from a nondifferentiated, aggressive, and proliferative state to a more differentiated and less aggressive state. The accumulation of GHB in GSCs has been shown to cause a decrease in 5-hydroxymethylcytosine (5-hmC) epigenetic mark via the repression of ten-11 translocation (TET) activity, which resulted in inhibition of proliferation, self-renewal, and stem cell marker expression of GSCs. Besides, GHB has also been shown to stimulate GSC cell adherence and generation of membrane extensions.^69^ Moreover, PN glioblastoma cells have high expression of TET1 and increased level of 5-hmC compared to mesenchymal cells, and TET1-mediated increase of 5-hmC is critical for PN cell tumorigenicity.^70^ Considering this, our finding of GHB enrichment in PN sEVs might suggest that 5-hmC-dependent PN cells secrete GHB, a TET antagonizing metabolite, via sEVs not only to maintain their cellular characteristics and tumorigenicity but also to support the invasive nature of surrounding MES cells.

Glioblastoma cells have been shown to express sugar transporters to scavenge galactose from the extracellular space and metabolize galactose by means of the Leloir and pentose phosphate pathways, allowing them to use an alternative source of energy.^71^ Our metabolic profiling also unveiled the presence of d-glucose and myo-inositol both in PN and MES sEVs, supporting the hypothesis that GSCs fuel their highly active biosynthesis and energy metabolism not only by expressing solute carrier transporters but also by taking up sEVs released from other tumor cells.

The metabolite screening also revealed that both PN and MES GSCs were enriched in most l-amino acids in comparison to their corresponding sEVs. Until now, only Cuperlovic-Culf et al. have revealed the metabolic profile of glioblastoma-derived extracellular vesicles and indicated that sEVs isolated from established glioblastoma cell lines, A172 and LN18, were enriched in tryptophan relative to their parental cells. Unlike A172-sEVs, LN18-derived sEVs were also found to be high in glycine, threonine, and homoserine.^72^ In our study, we captured only a few l-amino acids in our sEVs, and biological and technical reasons behind this observation remain elusive. To the best of our knowledge, no other studies on the total metabolite and amino acid content of GSC-derived sEVs have been carried out; therefore, in addition to our current work, further studies are needed to elaborate whether GSC-derived sEVs harbor l-amino acids. Knowing that glioblastoma cells are metabolically highly active and that many amino acids are critical for glioblastoma metabolism, it can be expected that GSCs preferentially retain amino acids and avoid their loading into sEVs. On the other hand, the sEV isolation and metabolite screening methods used in our study might also be responsible for low/no detection of amino acids in sEVs; therefore, different sEV enrichment techniques, such as size exclusion chromatography, immunoaffinity, and ultrafiltration, and metabolite identification methods should be tested in future studies to understand whether GSC-derived sEVs contains amino acids.

In addition to proteins and metabolites, there is emerging evidence of a tumor-promoting role for fatty acid metabolism in different cancer entities including glioblastoma.^13,73−78^ In this study, we showed that GSC-derived sEVs are rich in saturated fatty acids, whereas their parental cells were enriched in monounsaturated and polyunsaturated fatty acids, implying that loading of saturated fatty acids into sEVs may be a tightly regulated process. Some saturated fatty acids have been implicated in a variety of cancers;^77−82^ however, the biological, functional, and structural reasons behind their loading into sEVs and their role in glioblastoma are poorly defined and need further investigation.

Finally, we also found that cholesterol was enriched in GSC-derived sEVs, which provides precious insights into previous findings that de novo biosynthesis of cholesterol is suppressed in glioblastoma cells and that the cells are highly dependent on exogenous uptake of cholesterol for survival.^83^ SEV-mediated cholesterol transfer could imply the establishment of a metabolic cooperation between GSC populations and surrounding tumor cells, which rely exogeneous cholesterol uptake. Hence, blocking sEV-mediated transfer of cholesterol might be considered as an effective strategy for glioblastoma.

## Study Limitations

While our study provides in-depth characterization of the content of GSC-derived sEVs, technical limitations related to the detection of metabolites and fatty acids could mean that molecules present only at low levels have not been documented in this study. Further, the intense effort required to isolate large volumes of sEVs required for this multifaceted profiling means that our analysis is restricted to a relatively small number of patient-derived cell lines, which may limit the generalizability of the results. Finally, although we show that treating cells of the proneural subtype with sEVs or conditioned medium leads to transcriptional and proteomic changes *in vitro*, our data do not provide evidence for the physiological relevance of EVs in glioblastoma heterogeneity and plasticity. However, aside from the well-documented presence of sEVs in the serum of glioblastoma patients indicating a role for sEVs in glioblastoma biology, a similar study to ours, focusing solely on the protein content of GB sEVs, showed that PN glioblastoma cells mixed with MES sEVs increased their proliferative capacity and aggressiveness in a xenograft mouse model.^22^ Therefore, we believe that sEVs can induce transcriptional and proteomic changes to glioblastoma cells that affect their intrinsic biological properties and that these changes are also applicable *in vivo*.

## Conclusion

In conclusion, our study provides insights into the complexity of GSC-derived sEVs by revealing their protein, metabolite, fatty acid, and cholesterol content. This, along with the transcriptomic and proteomic changes induced in GSCs after treatment with sEVs or conditioned medium, demonstrates the potential contribution of GSC-derived sEVs to the plasticity, heterogeneity, and aggressiveness of glioblastoma.

## Material and Methods

### Cell Culture

Primary glioblastoma stem-like cell lines NCH421k, NCH644, NCH441, NCH705, and NCH711d were derived from glioblastoma patients who underwent surgical resection according to the research proposals approved by the Institutional Review Board at the Medical Faculty of Heidelberg. Glioblastoma stem-like cells (GSCs) were grown on hydrophobic growth surface cell culture flasks (Sarstedt) in Dulbecco’s Modified Eagle’s Medium/Nutrient Mixture F-12 Ham (Merck Millipore, F4815) supplemented with 2% (v/v) B-27 minus Vitamin A (Life Technologies; 12587010), 20 ng/mL epidermal growth factor (Life Technologies; PHG0311), 20 ng/mL basic fibroblast growth factor (Biomol; 50361.50), and 1 μg/mL heparin (Sigma-Aldrich; H3149-10KU). GSC neurospheres were dissociated using Accutase (Sigma-Aldrich; A6964). All cells were cultured in a cell culture incubator at 37 °C with 5% CO_2_ and 95% humidity and routinely tested for mycoplasma contamination (GATC Biotech).

### Fractionation of Conditioned Medium

The conditioned medium was fractionated by differential centrifugation and filtration. In brief, the conditioned medium was centrifuged at 300*g* for 20 min at 4 °C to get rid of cells and cell debris (named complete conditioned medium, CCM). Subsequently, CCM was centrifuged at 2000*g* for 20 min at 4 °C (Heraeus Varifuge 3.OR), and the supernatant was collected (2000*g* supernatant, 2S). The 2000*g* supernatant was depleted at 10,000*g* for 20 min at 4 °C, and the pellet containing cell debris and medium/large vesicles was discarded. Afterward, the 10,000*g* supernatant (10S) was centrifuged at 100,000*g* for 2 h at 4 °C (SW40Ti, #331301, adjusted k-factor 388.81) to pellet the small extracellular vesicles (100P). The 100,000*g* supernatant (100S) containing secreted proteins was also stored on ice until use. Lastly, in addition to differential centrifugation, the complete conditioned medium was filtered with a 0.02 μm-membrane filter (GE Healthcare, #6809-2102) to eliminate the extracellular vesicles, sparing the soluble factors in the flow-through.

### Treating Proneural Cells with the Different Fractions of Mesenchymal Conditioned Medium

Proneural (PN) cells were treated with the different fractions of conditioned medium of mesenchymal (MES) cells, and the changes in abundance of well-known mesenchymal and proneural cell surface markers (CD44 and CD133, respectively) were measured using flow cytometry (BD LSRFortessa, BD Biosciences). As such, mesenchymal cells (NCH705 or NCH711d) were seeded into cell culture flasks (2.5 × 10^6^ cells in 12 mL of medium) and cultured for 4 days, and their conditioned medium was fractionated as described above. NCH421k (PN) cells were seeded in cell culture flasks at a density of 1.5 × 10^6^ cells in 6 mL of heparin-free medium, treated with 6 mL of different fractions (Own_CCM, MES_CCM, 2S, 10S, 100S and Filtered) of mesenchymal conditioned medium, and then cultured for 4 days prior to staining for cell surface markers. For the 100P fraction, sEVs containing pellet, obtained from 6 mL of media, were dissolved in 1 mL of heparin-free medium and transferred into a cell culture flask containing 1.5 × 10^6^ NCH421k cells in 11 mL of heparin-free medium. After the incubation, the cells were harvested by centrifugation, dissociated with Accutase (Sigma-Aldrich; A6964), and washed twice with DPBS (Sigma-Aldrich; D8537). The dissociated cells were stained with PE/Cy7 antihuman CD44 (Biolegend, #338816) and APC antihuman CD133 (Biolegend, #372805) antibodies in DPBS containing 5% fetal bovine serum (FBS) on ice for 30 min in the dark. Afterward, antibody-stained cells were treated with propidium iodide (Sigma-Aldrich, #P4864) at a final concentration of 0.5 μg/mL for 5 min to discriminate dead cells from viable cells. Control stainings were performed by replacing each primary antibody with their nonimmune isotypes PE/Cy7Mouse IgG1, κ or APC Mouse IgG1, κ (Biolegend, #400125 and #400121, respectively), at the same concentration, and used for setting the gates for flow cytometry analysis. Finally, the data were acquired by flow cytometry (BD LSRFortessa, BD Biosciences) and analyzed by FlowJo software (FlowJo-LLC, USA).

### Separation of Small Extracellular Vesicles (sEVs)

Small extracellular vesicles (sEVs) were separated using differential ultracentrifugation (dUC) as described previously with some modifications as follows.^84^ Glioblastoma stem-like cells were cultured in DMEM/F12 medium supplemented with 2% (v/v) B-27 minus Vitamin A, 20 ng/mL EGF, 20 ng/mL bFGF, and 1 μg/mL heparin for 3 days. Conditioned medium was harvested and centrifuged at 300*g* for 20 min to remove cells and debris. Subsequently, supernatant was collected and centrifuged at 10,000*g* for 20 min at 4 °C in a fixed-angle rotor (Sorvall SS-34, adjusted k-factor 3586.32) to eliminate large vesicles. Afterward, sEVs were pelleted by ultracentrifugation at 100,000*g* for 70 min at 4 °C in an SW28Ti swinging-bucket rotor (Beckman Coulter, #342204, adjusted k-factor 346.84) and resuspended in 0.22 μm-membrane filtered DPBS (Sigma-Aldrich; D8537). To increase the purity of separated sEVs, resuspended sEVs were loaded onto a 20% (w/v) iodixanol density cushion prepared by mixing 4 volumes of 50% (w/v) iodixanol working solution (5 volume of OptiPrep (Sigma-Aldrich; D1556) mixed with 1 volume of diluent containing 0.25 M sucrose, 6 mM ethylenediaminetetraacetic acid (EDTA), and 60 mM Tris-HCl, pH 7.4) with 6 volumes of homogenization media (0.25 M sucrose, 1 mM EDTA, 10 mM Tris-HCl, pH 7.4) and centrifuged again at 100,000*g* for 70 min at 4 °C in an SW40Ti swinging-bucket rotor (Beckman Coulter, #331301, adjusted k-factor 388.81). After centrifugation, the white band containing the sEVs was collected in a total volume of 2 mL and washed with 8 mL of 0.22 μm-membrane filtered DPBS. Finally, sEVs were pelleted by ultracentrifugation at 100,000*g* for 70 min at 4 °C using an SW40Ti swinging-bucket rotor (Beckman Coulter, #331301, adjusted k-factor 388.81), resuspended in 25–50 μL of 0.22 μm-membrane filtered ice-cold DPBS, and stored at −80 °C in microcentrifuge tubes. Heraeus Varifuge 3.OR and Sorvall RC 5B PLUS centrifuges were used for the initial centrifugation steps (300 and 10,000*g*, respectively), and subsequent steps were conducted in a Beckman Coulter L8–70 M ultracentrifuge. All spins were performed with maximum acceleration and deceleration.

### Nanoparticle Tracking Analysis (NTA)

The concentration and size distribution of separated sEVs were profiled by nanoparticle tracking analysis (NTA) using a NanoSight LM10 system (Malvern Instruments, Worcestershire, UK) equipped with a blue laser (405 nm laser) and an sCMOS camera following the manufacturer’s guidelines. Samples were first diluted (1:250 for NCH421k sEVs and 1:500 for NCH644, NCH705, and NCH711d sEVs) in 0.22 μm-membrane filtered DPBS (Sigma-Aldrich, D8537), and the particles were tracked by using the following settings: Camera Level: 8, Slider Shutter: 350, Slider Gain: 250, FPS: 25.0, Temperature 23.7 °C ± 0.4 °C. Five 60-s videos were recorded for each sample and analyzed using the NTA 3.0 software (Malvern Instruments, Worcestershire, UK) with a detection threshold of 5.

### Transmission Electron Microscopy (TEM)

Separated small extracellular vesicles (sEVs) were adsorbed onto glow discharged carbon-coated copper grids, washed with bidistilled water followed by negative staining with 2% aqueous uranyl acetate. Electron micrographs were taken with a Zeiss EM 912 at 120 kV (Carl Zeiss, Oberkochen, Germany) using a slow scan charge-coupled device (CCD) camera (TRS, Moorenweis, Germany).

### Western Blotting

For protein extraction from GSCs, pelleted cells were first lysed with RIPA Buffer (Abcam, #ab156034), loaded into QIAShredder columns (Qiagen, #79656), and centrifuged at 13,000 rpm for 2 min, and the flow-through containing proteins was collected. To extract the proteins from sEVs, 2% sodium dodecyl sulfate (SDS) was mixed with the samples (1:9, v/v), vortexed for 30 s, and incubated at room temperature for 10 min. Afterward, samples were centrifuged at 11,000*g* for 10 min, and the protein-containing supernatant was carefully recovered. For Western blotting, an equal amount of protein (5 μg) from cell lysates was prepared in lithium dodecyl sulfate (LDS) Sample Buffer (Invitrogen, NP0007) supplemented with reducing agent dithiothreitol (DTT) (Invitrogen, NP0009) and denatured at 95 °C for 5 min. In addition, sEV samples were prepared by mixing 5 μg of sEV proteins with 2% SDS (at final a concentration of 0.2%, w/v), LDS Sample Buffer (Invitrogen, NP0007), and DTT (Invitrogen, NP0009), followed by denaturation at 70 °C for 10 min. Boiled protein samples were loaded into the wells of 4%–12% NuPAGE Bis-Tris gel (Invitrogen, NP0321BOX) and electrophoresed at 250 V, 170 mA for 45 min in NuPAGE MES SDS Running Buffer (Invitrogen, NP0002) containing antioxidant (Invitrogen, NP0005). Afterward, the gel was carefully placed in transfer buffer (25 mM Tris, 200 mM glycine, 15% isopropanol, pH 8.8), and the separated proteins were transferred onto a methanol-activated poly(vinylidene difluoride) (PVDF) membrane (Merck Millipore, IPVH00010) by performing a wet transfer in a Mini Trans-Blot Cell (Bio-Rad) wet gel transfer system, increasing the current 100 mA every 10 min for 50 min. Subsequently, the blot was blocked in blocking buffer (5% w/v BSA/Milk in TBST (0.1% Tween 20)) at room temperature for 1 h and probed with primary antibodies against CD138 (Biolegend, #352302, 1:200), ALIX (Cell Signaling, #2171, 1:1000), ENO1 (Abcepta, #AP6526c, 1:500), TSG101 (GeneTex, #GTX70255, 1:500), CD9 (Cell Signaling, #13403, 1:1000), GM130 (Cell Signaling, #12480, 1:1000), Lamin-B1 (Abcam, #ab16048, 1:5000), RPLP0 (Atlas Antibodies, #HPA003512, 1:250), Cytochrome C (Biolegend, #612503, 1:500), ANXA1 (Cell Signaling, #32934S, 1:1000), Thy1 (Cell Signaling, #13801S, 1:500), CD133 (PROM1, Cell Signaling, #5860S), ITGA5 (Cell Signaling, #4705T, 1:1000), and α-Tubulin (Sigma-Aldrich, #T9026, 1:10,000) at 4 °C on a roller overnight. The membrane was rinsed three times in TBST and incubated in with horseradish peroxidase-conjugated secondary antibodies (Cell Signaling, Mouse #7076S, Rabbit #7074S, 1:5000) for 2 h at room temperature on a roller. Finally, the membrane was rinsed three times with TBST and incubated with chemiluminescent substrate (Thermo Fisher Scientific, #32106) according to manufacturer’s protocol. The blots were placed in a cassette, and chemiluminescent signal was captured by exposing the blots to X-ray films (Super RX-N, Fujifilm). The developed films were scanned using a Ricoh MP C4504ex scanner.

### Monitoring sEV Uptake Using Confocal Microscopy and Flow Cytometry

In order to visualize sEV uptake by confocal microscopy, fluorescent lipophilic dye PKH26 (Sigma-Aldrich; MINI26, 1:50) stained or genetically labeled (PalmtdTomato) sEVs were used. The pelleted sEVs were first stained with PKH26 at room temperature for 30 min in the dark. After incubation with the staining solution, sEVs were washed with 0.22 μm-membrane filtered DPBS, loaded onto iodixanol density cushion (OptiPrep, Sigma-Aldrich; D1556), and centrifuged at 100,000*g* for 70 min at 4 °C (SW40Ti, #331301, adjusted k-factor 388.81). Subsequently, stained sEVs were carefully collected, washed with DPBS, and centrifuged again at 100,000*g* for 70 min at 4 °C. The pelleted sEVs were resuspended in filtered DPBS. In addition, PalmtdTomato labeled sEVs were separated as described above from NCH705 cells, which were stably transduced with a lentivirus vector expressing PalmtdTomato, tandem dimer Tomato fused at NH_2_-termini with a palmitoylation signal. The internalization of PKH26 stained or PalmtdTomato tagged sEVs by recipient cells was visualized using a Leica TCS SP5 confocal microscope (Leica Microsystems, Wetzlar, Germany).

To measure sEV uptake using flow cytometry, NCH705 cells were stably transduced with a PalmtdTomato-expressing lentiviral vector, and their sEVs (PalmtdTomato labeled) were separated as described above. PalmGFP expressing NCH421k cells (recipient) were seeded into chambered coverslips (Ibidi, μ-Slide 8 Well; #80826) at a density of 10,000 cells per well and treated with PalmtdTomato-tagged NCH705 sEVs or DPBS (control), and the uptake of labeled sEVs uptake was measured every 2 h for 8 h using a BD LSRFortessa (BD Biosciences) flow cytometer. The data were analyzed using FlowJo software (FlowJo-LLC, USA).

### Proteomics of Glioblastoma Stem-Like Cells and Their sEVs

#### Sample Preparation

Five × 10^6^ cells (NCH421k, NCH644, NCH705, and NCH711d) were seeded into each of 12 T175 cell culture flasks (Sarstedt, 83.3912.502) containing 25 mL of complete DMEM/Ham’s F12 medium and allowed to grow for 3 days. Subsequently, conditioned medium was harvested from each cell culture flask and pooled for sEV separation as described above. Additionally, sEV-producing GSCs were recovered after the 300*g* centrifugation step, washed with DPBS (Sigma-Aldrich, D8537), and dissociated into single cells with Accutase (Sigma-Aldrich, A6964) for 3 min at 37°. Cells were counted with a Vi-CELL XR Cell Viability Analyzer (Beckman Coulter); 1 × 10^7^ cells were snap-frozen in liquid nitrogen and stored at −80 °C until use. To extract proteins from sEVs, samples were lysed in RIPA Buffer (Abcam, #ab156034) for 10 min and centrifuged at 11,000 rpm for 10 min, and the protein-containing supernatant was collected. For protein extraction from cells, samples were lysed in RIPA Buffer (Abcam, #ab156034) for 10 min, loaded into QIAShredder columns (Qiagen, #79656) and centrifuged at 13,000 rpm for 2 min, and the flow-through containing proteins was carefully collected. Afterward, proteins (10 μg) were run for 0.5 cm into an SDS-polyacrylamide gel electrophoresis (PAGE), and the gel pieces containing the sample were cut out after Coomassie staining and used for subsequent digestion using trypsin according to Shevchenko et al.^85^ adapted to the DigestPro MSi robotic system (INTAVIS Bioanalytical Instruments AG).

#### MS Method Setup

LC-MS/MS analysis of sEVs was carried out on an Ultimate 3000 UPLC system (Thermo Fisher Scientific) connected to an Orbitrap Exploris 480 mass spectrometer (Thermo Fisher Scientific). Total LC-MS/MS analysis time was 90 min per sample. Prior to the analytical separation, peptides were online desalted on a trapping cartridge (Acclaim PepMap300 C18, 5 μm, 300 Å pore; Thermo Fisher Scientific) for 3 min using 30 μL/min flow of 0.05% trifluoroacetic acid (TFA) in water. The analytical multistep gradient was carried out on a nanoEase MZ Peptide analytical column (300 Å, 1.7 μm, 75 μm × 200 mm, Waters) using solvent A (0.1% formic acid in water) and solvent B (0.1% formic acid in acetonitrile). The concentration of solvent B was linearly ramped from 4% to 30% in 72 min, followed by a quick ramp up to 78% B. After 2 min, the concentration of solvent B was lowered back to 2%, and a 10 min equilibration step was appended. Eluting peptides were analyzed in the mass spectrometer using data-dependent acquisition (DDA) mode. A full scan at 60k resolution (380–1400 *m*/*z*, 300% AGC target, 45 ms maxIT) was followed by 1.5 s of MS/MS scans. Peptide features were isolated with a window of 1.4 *m*/*z* and fragmented using 26% normalized collision energy (NCE). Fragment spectra were recorded at 15k resolution (100% AGC target, 54 ms maxIT). Unassigned and singly charged eluting features were excluded from fragmentation, and dynamic exclusion was set to 35 s. LC-MS/MS analysis of whole cell lysates was performed as described above with the following changes: Total LC-MS/MS time was 150 min per sample during which the concentration of B was linearly ramped from 4%–30% within 134 min. A full scan at 120k resolution (380–1400 *m*/*z*, 300% AGC target, 45 ms maxIT) was followed by up to 2 s of MS/MS scans. Fragment spectra were recorded at 15k resolution (100% AGC target, 22 ms maxIT). Unassigned and singly charged eluting features were excluded from fragmentation, and dynamic exclusion was set to 35 s.

#### Data Analysis and Statistics

Data analysis was carried out by MaxQuant version 1.6.14.0^86^ using an organism-specific database extracted from Uniprot.org under default settings (human containing 74811 entries from 27.02.2020). Identification FDR cutoffs were 0.01 on peptide level and 0.01 on protein level. The match between runs (MBR) option was enabled to transfer peptide identifications across RAW files based on accurate retention time and *m*/*z*. For the analysis, fractions were set in a way that MBR was only performed within each condition. LFQ quantification was done using a label-free quantification approach based on the MaxLFQ algorithm.^87^ A minimum of 2 quantified peptides per protein was required for protein quantification. “Separate LFQ in parameter groups” was enabled so an individual LFQ normalization was performed on the three parameter groups “cell lysates”, “sEVs”, and “mock sEVs isolation”. In addition, iBAQ-values^88^ were generated via MaxQuant. For “sEV to cell” and “cell to cell” (or “sEV to sEV”) comparisons, iBAQ and LFQ values were used, respectively.

The following filtering, normalization, and imputation were performed individually for each statistical contrast: Adapted from the Perseus recommendations,^89^ protein groups with valid LFQ or iBAQ values in 70% of the samples of at least one of the conditions were used for statistics. LFQ values were normalized via variance stabilization normalization.^90^ In addition, adapted from the Perseus recommendations,^89^ missing LFQ or iBAQ values (that are completely absent in one condition) were imputed with random values drawn from a downshifted (2.2 standard deviation) and narrowed (0.3 standard deviation) intensity distribution of the individual samples. For partially missing LFQ or iBAQ values (partial absence in one condition), the R-package missForest was used for imputation.^91^ The statistical analysis for LFQ and iBAQ values was performed with the R-package “limma”.^92^ The p-values were adjusted with the Benjamini–Hochberg method for multiple testing.^93^

Differentially abundant proteins were identified (i) for the comparisons of sEVs to cell lysates in individual cell lines using the criteria of adjusted p-value <0.001 and absolute log_2_-fold change cutoff >4 and (ii) for the comparisons of sEVs between PN and MES subtypes using the threshold of adjusted p-value <0.05 and absolute log_2_-fold change >1. For all the pairwise comparisons, functional enrichment analysis was performed with the R-package “clusterProfiler” (v4.2.1) to identify enriched pathways/processes from Gene Ontology (GO) and Kyoto Encyclopedia of Genes and Genomes (KEGG) annotations among the differentially abundant proteins. R fgsea package (v1.20.0, fgseaMultilevel function with parameter eps = 0)^94^ was employed for gene set enrichment analysis^95^ (GSEA, preranked) on the resulting log_2_-fold change values of the differential analysis. Enriched GSEA terms were visualized by R package enrichplot (v1.18.3, gseaplot2 function).^96^ Gene set enrichment analysis was carried out using the reference annotation of MSigDB database (v7.5.1).^97^ Finally, the UpSet plot was generated using UpSetR version 1.4.0,^98^ and the volcano plots were created by VolcaNoseR web app.^99^

### RNA-Sequencing and Mass-Spectrometry of PN Cells Treated with MES CM or sEVs

#### Sample Preparation and Extraction

750,000 PN GSCs were seeded in 3 mL of GSC medium and treated with 3 mL of conditioned medium (CM) obtained from MES GSCs grown for 4 days. Four days later, cells were harvested, dissociated with Accutase (Sigma-Aldrich, A6964), and washed with DPBS (Sigma-Aldrich, #D8537). Each sample was divided into two parts for RNA and protein isolation. For EV treatment, 250,000 PN cells were incubated with NCH705-derived sEVs (9 μg protein equivalent) for 4 days and harvested, dissociated, and washed for protein extraction. To extract proteins, cell pellets were resuspended in 50 μL of RIPA Buffer (Abcam, #ab156034) and incubated for 15 min on ice. Lysates were homogenized using QIAShredder columns (Qiagen, #79656), and protein concentration was determined by BCA assay (Thermo Fisher Scientific, #23225). RNA was isolated using an RNeasy mini kit (Qiagen, #74104) according to the manufacturer’s protocol.

#### Sequencing and Mass Spectrometry

Sequencing libraries were prepared using the Illumina TruSeq mRNA stranded Kit following the manufacturer’s instructions. Briefly, mRNA was purified from 500 ng of total RNA using oligo(dT) beads. Then poly(A)^+^ RNA was fragmented to 150 bp and converted to cDNA. The cDNA fragments were then end-repaired, adenylated on the 3′ end, adapter ligated, and amplified with 15 cycles of polymerase chain reaction (PCR). The final libraries were validated using Qubit (Invitrogen) and Tapetstation (Agilent Technologies). 2× 100 bp paired-end sequencing was performed on the Illumina NovaSeq 6000 according to the manufacturer’s protocol. The LC-MS/MS analysis of whole cell lysates was performed as described above in section “*MS method setup*”.

#### Data Analysis and Statistics

Differential expression analysis was carried out by using the Bioconductor package of edgeR^100^ with the recommended functions of estimateDisp, glmQLFit, and glmQLFTest and a paired sample design (n = 3 paired replicates). P-values were adjusted by the Benjamini-Hochberg method to correct for multiple testing.^93^ GSEA-preranked analysis was performed based on the fold change of all expressed genes.^95,100^ For proteomics, data analysis was carried out as described above in section “*Data analysis and statistics*”.

### Metabolite Screening and Fatty Acid Profiling by Gas Chromatography–Mass Spectrometry (GC-MS)

#### Sample Preparation and Extraction

GSCs were seeded into each of 12 T175 cell culture flasks (Sarstedt, 83.3912.502) at a density of 5 × 10^6^ cells/25 mL of complete DMEM/Ham’s F12 medium and cultured for 3 days. Afterward, conditioned medium was collected from each cell culture flask and pooled for sEV separation as described above. In addition, sEV-producing GSCs were also harvested after the 300*g* centrifugation step, washed with DPBS (Sigma-Aldrich, D8537), dissociated using Accutase (Sigma-Aldrich, A6964) for 3 min at 37°, and counted with Vi-CELL XR Cell Viability Analyzer (Beckman Coulter). Next, 1 × 10^7^ cells were snap-frozen in liquid nitrogen and stored at −80 °C until use. For metabolite and fatty acid extraction, samples were treated with 380 μL of methanol supplemented with 0.2 mg/mL ribitol at 70 °C for 15 min. Afterward, 200 μL of chloroform containing 20 mg/mL heptadecanoic acid (C17:0) was added, and samples were shaken at 37 °C for 5 min. Subsequently, 400 μL of water was added, and the samples were centrifuged at 11,000*g* for 10 min to separate polar and organic phases. For the derivatization, 700 μL of the polar phase (upper phase) was carefully transferred into a fresh GC vial and dried using an Eppendorf Concentrator Plus without heating. To analyze total fatty acids, 150 μL of the lower organic phase (chloroform) was transferred into fresh vials and dried in a speed vacuum without heating. The protein phase was used for the normalization.

#### Derivatization

Sequential online methoximation and silylation reactions were conducted with MPS autosampler (Gerstel GmbH & Co. KG) for the gas chromatographic (GC) screening of metabolites. Methoximation was carried out by treating each sample with 20 μL of 20 mg/mL methoxyamine hydrochloride (Sigma-Aldrich) in pyridine (Sigma-Aldrich) at 37 °C for 90 min in a Gerstel MPS Agitator Unit (250 rpm). Afterward, for the silylation reactions, the samples were treated with 45 μL of *N*-methyl-*N*-trimethylsilyl-trifluoroacetamide (Sigma-Aldrich) supplemented with C4–C24 Fatty Acid Methyl Ester (FAME) Standards (1 μg/mL) and incubated at 37 °C for 30 min with gentle shaking. Before the injection, samples were incubated at room temperature for 45 min. For the fatty acid analysis, sequential online transmethylation reactions were performed using an MPS autosampler (Gerstel GmbH & Co. KG). Briefly, the pellets were redissolved in 40 μL of TBME (*tert*-butyl methyl ether, Sigma-Aldrich) for 5 min at 500 rpm at 50 °C and incubated with 20 μL of TMSH (trimethylsulfonium hydroxide, Sigma-Aldrich) for 45 min at 500 rpm at 50 °C.

#### Gas Chromatography/Mass Spectrometry (GC/MS) Analysis

For the GC screening of metabolites, a GC-TOF system (Agilent 7890 GC; Rxi-5Sil MS Columns; Pegasus BT) was used for the gas chromatography/mass spectrometry (GC/MS) analysis, and data processing was performed with ChromaTOF v5.50 software. The GC was operated at an injection temperature of 250 °C, and 1 μL of sample was injected in splitless mode for the small extracellular vesicles and with a split ratio of 1:10 for the cells using the following conditions: 1 min hold at 40 °C; 6 °C/min ramp to 210 °C; 20 °C/min ramp to 330 °C; bake-out at 330 °C for 5 min using helium as a carrier gas with constant linear velocity. Additionally, the ion source and interface temperatures were set at 250 °C with a solvent cut time of 8.5 min, a scan range (*m*/*z*) of 50–600, and an acquisition rate of 17 spectra/second. For fatty acid profiling, GC/MS-QP2010 Plus (Shimadzu) fitted with a Zebron ZB 5MS column (Phenomenex; 30 m × 0.25 mm × 0.25 μm) was used in the GC/MS analysis. The GC was operated with an injection temperature of 230 °C, and 1 μL of sample was injected in splitless mode for the small extracellular vesicles and with a split ratio of 1:5 for cell samples using the following conditions: 1 min hold at 40 °C; 6 °C/min ramp to 210 °C; 20 °C/min ramp to 330 °C; bake-out at 330 °C for 5 min using helium as a carrier gas with constant linear velocity. The mass spectrometer was operated with ion source and interface temperatures of 250 °C, solvent cut time of 7 min, and a scan range (*m*/*z*) of 40–700 with an event time of 0.2 s. The GC/MS solution software (Shimadzu) was used for data processing.

#### Data Processing and Analysis

The raw peak area values were normalized to the internal standards ribitol and heptadecanoic acid (C17:0) in the GC screening and fatty acid profiling, respectively. The values obtained from the extraction blank samples were subtracted from all sample values. Besides, normalized values measured in the control sample (mock sEVs isolation from cell-free conditioned medium) were subtracted from each sEV sample to identify the metabolites and fatty acids truly associated with the sEVs.^101^ The metabolomics and fatty acid data were further normalized to per μg of protein isolated from the cells and sEV samples using the protein phase formed in the sample extraction step. Finally, hierarchical clustering heatmaps of metabolites and fatty acids, metabolite set enrichment analysis (MSEA) plots, sparse partial least-squares-discriminant analysis (sPLS-DA) plot, correlation heatmap of fatty acids, and variable importance in projection (VIP) score plots were generated using MetaboAnalyst 5.0.^102^ Box and whisker plots were generated using GraphPad Prism 7 (GraphPad Software, San Diego, California USA).

### Statistical Analysis

GraphPad Prism 7 (GraphPad Software, San Diego, California USA) was used to perform ratio paired *t* test and unpaired *t* tests. Statistical significance was indicated as follows: * *p* < 0.05, ** *p* < 0.01, *** *p* < 0.001, **** *p* < 0.0001, ns: not significant.

## Data Availability

We have submitted all relevant data of our experiments to the EV-TRACK knowledgebase^103^ with EV-TRACK ID: EV220326. The mass spectrometry proteomics data have been deposited in the ProteomeXchange Consortium via the PRIDE partner repository^104^ with the data set identifier PXD036359.

## Supporting Information Available

The proteins significantly enriched in PN/MES sEVs (XLSX)
Full names of metabolites and their normalized (per μg of protein) intensities (XLSX)
Figures S1–S11 including flow cytometry results of PN cells treated with the conditioned medium of MES cells, qualitative analysis and internalization of GSC-derived sEVs, proteome profiling of GSC-derived sEVs and their parental cells, volcano plots of quantified proteins in sEVs and WCLs, GO enrichment analysis of proteins enriched in sEVs in comparison to their respective GSC lines, upset plot indicating specific and shared proteins found in our and Ricklefs GSC-derived sEVs proteome data set, GO enrichment analysis of proteins enriched in MES or PN sEVs, RNA-sequencing and mass-spectrometry based analysis of PN cells upon the treatment with MES CM/sEVs, metabolites identified in PN/MES GSCs and MSEA results of PN/MES sEVs, box plots of the free fatty acids that are significantly low in PN and/or MES sEVs and variable importance in projection (VIP) score plots (PDF)

## Abbreviations

GSCs: glioblastoma stem-like cells
PN: proneural
MES: mesenchymal
sEVs: small extracellular vesicles
LC-MS/MS: liquid chromatography–mass spectrometry
GC-MS: gas chromatography–mass spectrometry
FFA: free fatty acids
