# Supplementary material for: Proteomic, Metabolomic, and Fatty Acid Profiling of Small Extracellular Vesicles from Glioblastoma Stem-Like Cells and Their Role in Tumor Heterogeneity
Source: ACS Nano. 2024 Jan 11;18(3):2500–19. doi: 10.1021/acsnano.3c11427 (PMC10811755; doi:10.1021/acsnano.3c11427)
Supplement: Supplementary file 3 — nn3c11427_si_003.pdf [file nn3c11427_si_003.pdf]

# SUPPORTING INFORMATION

# Proteomic, metabolomic, and fatty acid profiling of small extracellular vesicles from glioblastoma stem-like cells and their role in tumor heterogeneity

Tolga Lokumcu,[▲,§] Murat Iskar,[‡] Martin Schneider,[†] Dominic Helm,[†] Glynis Klinke,[•] Lisa Schlicker,[†,∮] Frederic Bethke,[▲] Gabriele Müller,[▲] Karsten Richter,[#] Gernot Poschet,[•] Emma Phillips,[*,▲] Violaine Goidts[*,▲]

[▲] Brain Tumor Translational Targets, German Cancer Research Center (DKFZ), Heidelberg 69120, Germany.

[§] Faculty of Biosciences, University of Heidelberg, Heidelberg 69120, Germany.

[‡] Friedrich Miescher Institute for Biomedical Research, Basel 4058, Switzerland.

[†] Proteomics Core Facility, German Cancer Research Center (DKFZ), Heidelberg 69120, Germany.

[•] Metabolomics Core Technology Platform, Centre for Organismal Studies, Heidelberg University, Heidelberg 69120, Germany.

[∮] Division of Tumor Metabolism and Microenvironment, German Cancer Research Center (DKFZ), Heidelberg 69120, Germany

[#] Core Facility Electron Microscopy, German Cancer Research Center (DKFZ), Heidelberg 69120, Germany.

*Correspondence: v.goidts@dkfz.de; e.phillips@dkfz.de

Supplementary Figure S1

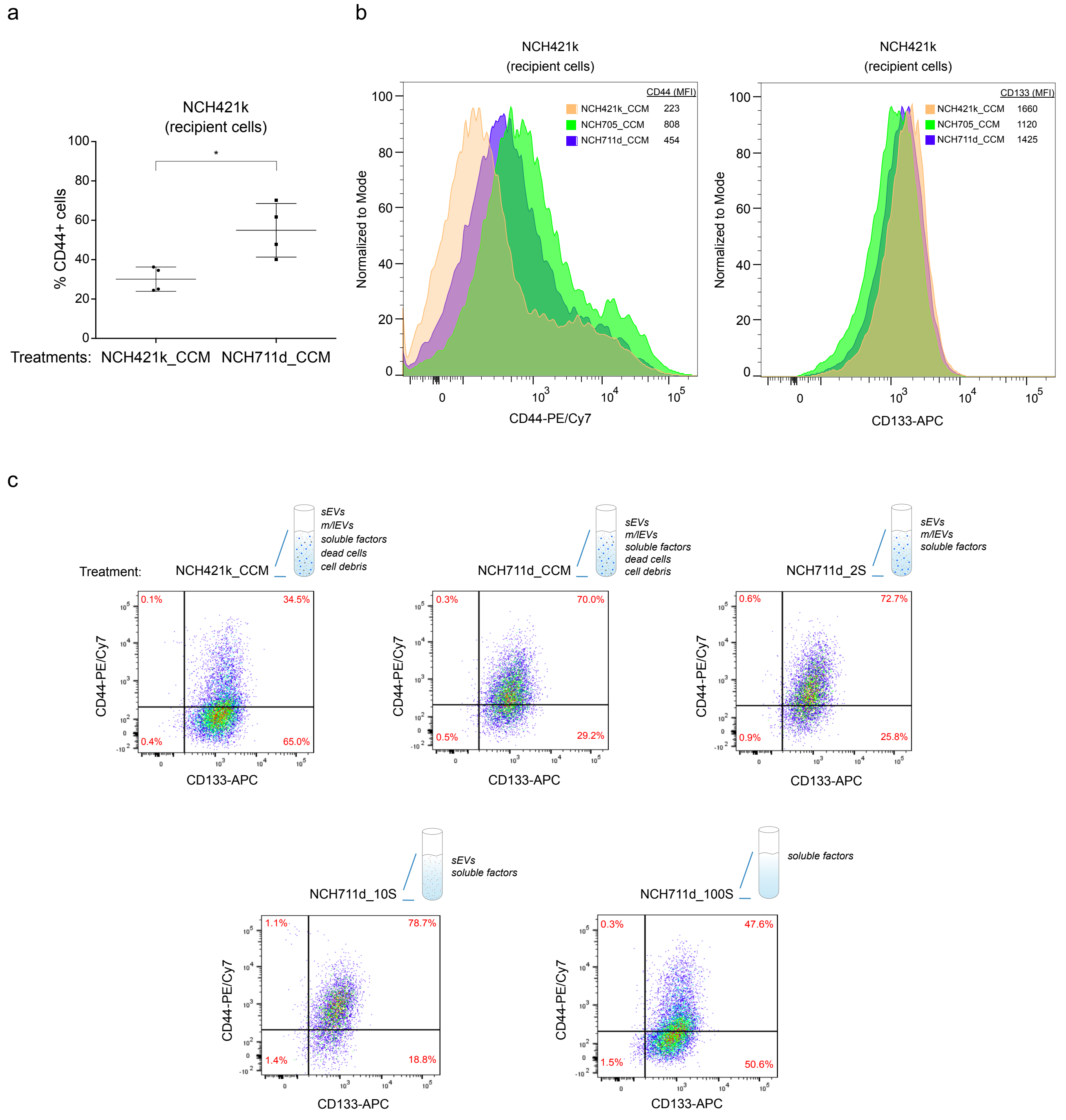


**Supplementary Figure S1.** Flow cytometry results of PN cells treated with the conditioned medium of MES cells. (a) NCH421k cells (PN) were treated with the complete conditioned medium of mesenchymal NCH711d cells, and percent CD44-positivity was measured by flow cytometry. *$p<0.05$, as determined by ratio paired t-test. Mean with SD of n=4 biological replicates is shown. (b) PN NCH421k cells were treated with the complete conditioned medium (CCM) of MES NCH705 (NCH705_CCM) and NCH711d cells (NCH711d_CCM), and the abundance of CD44 and CD133 (MES and PN cell markers, respectively) was measured by flow cytometry. NCH421k cells treated with their own complete condition medium (NCH421k_CCM) were used as a control. Histograms are showing the mean fluorescent intensity (MFI) of CD44 (left) and CD133 (right) upon treatment. (c) Flow cytometry results displaying CD44 and CD133 positive PN NCH421k cells upon treatment with the different fractions of MES NCH711d cells. NCH421k_CCM, NCH421k complete conditioned medium; NCH711d_CCM, NCH711d complete conditioned medium; NCH711d_2S/10S/100S, NCH711d conditioned medium fractions (supernatant) obtained after centrifugation at 2000 g/10,000 g/100,000 g.

Supplementary Figure S2

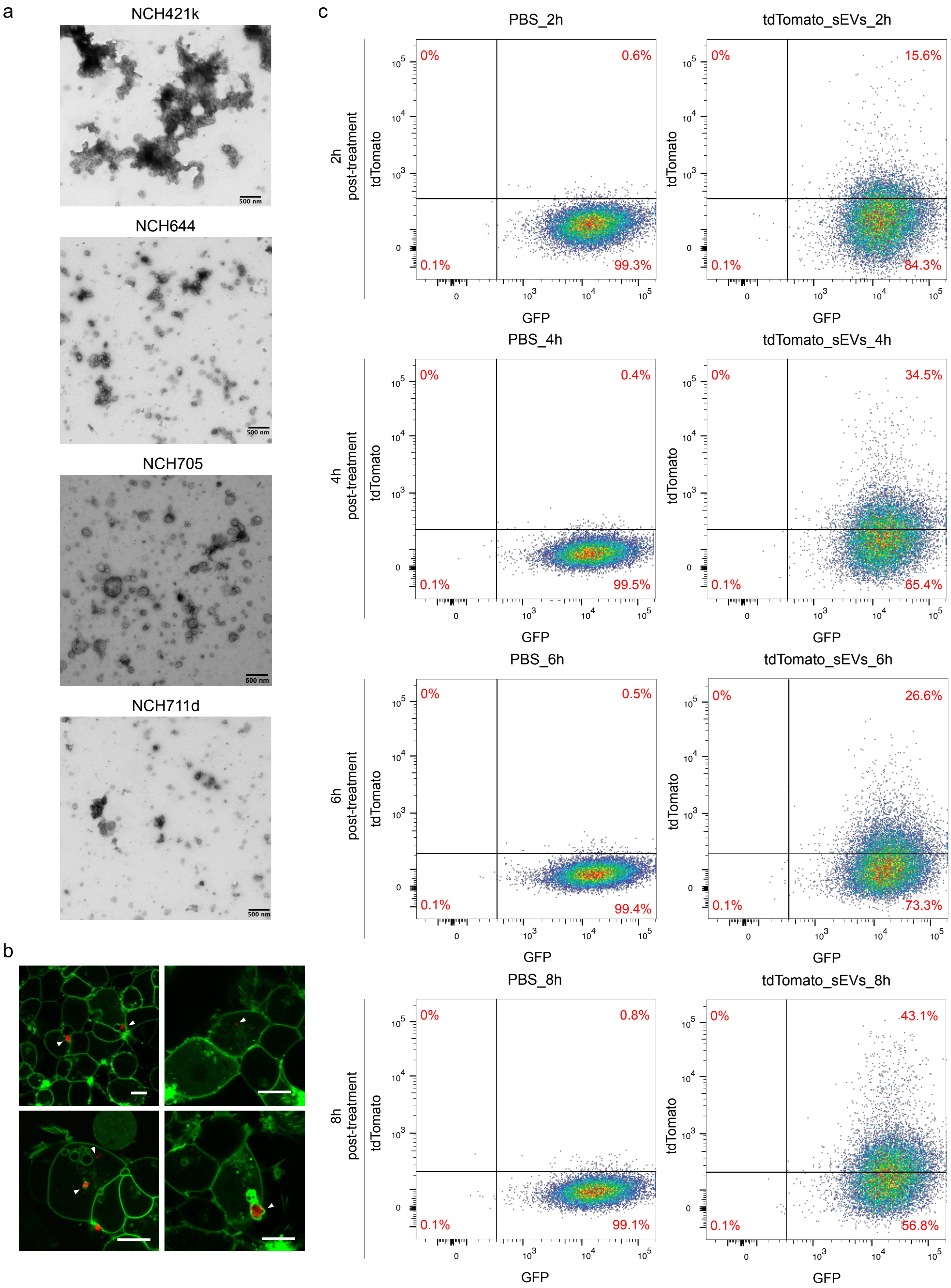


**Supplementary Figure S2.** Qualitative analysis and internalization of GSC-derived sEVs. (a) Wide-field transmission electron microscopy (TEM) micrographs of NCH421k, NCH644, NCH705, and NCH711d sEVs. Scale bar, 500 nm. (b) Representative confocal micrographs showing the internalization of PalmtdTomato tagged NCH705 sEVs (arrowhead) by NCH421k-PalmGFP cells. Scale bar, 10 μm. (c) Flow cytometry results showing time-course uptake of sEVs. NCH421k-PalmGFP cells (recipient) were treated with the sEVs isolated from NCH705-PalmtdTomato cells (donor), and sEV uptake was monitored every 2 hours for 8 hours. PBS treated cells were used as a control.

Supplementary Figure S3

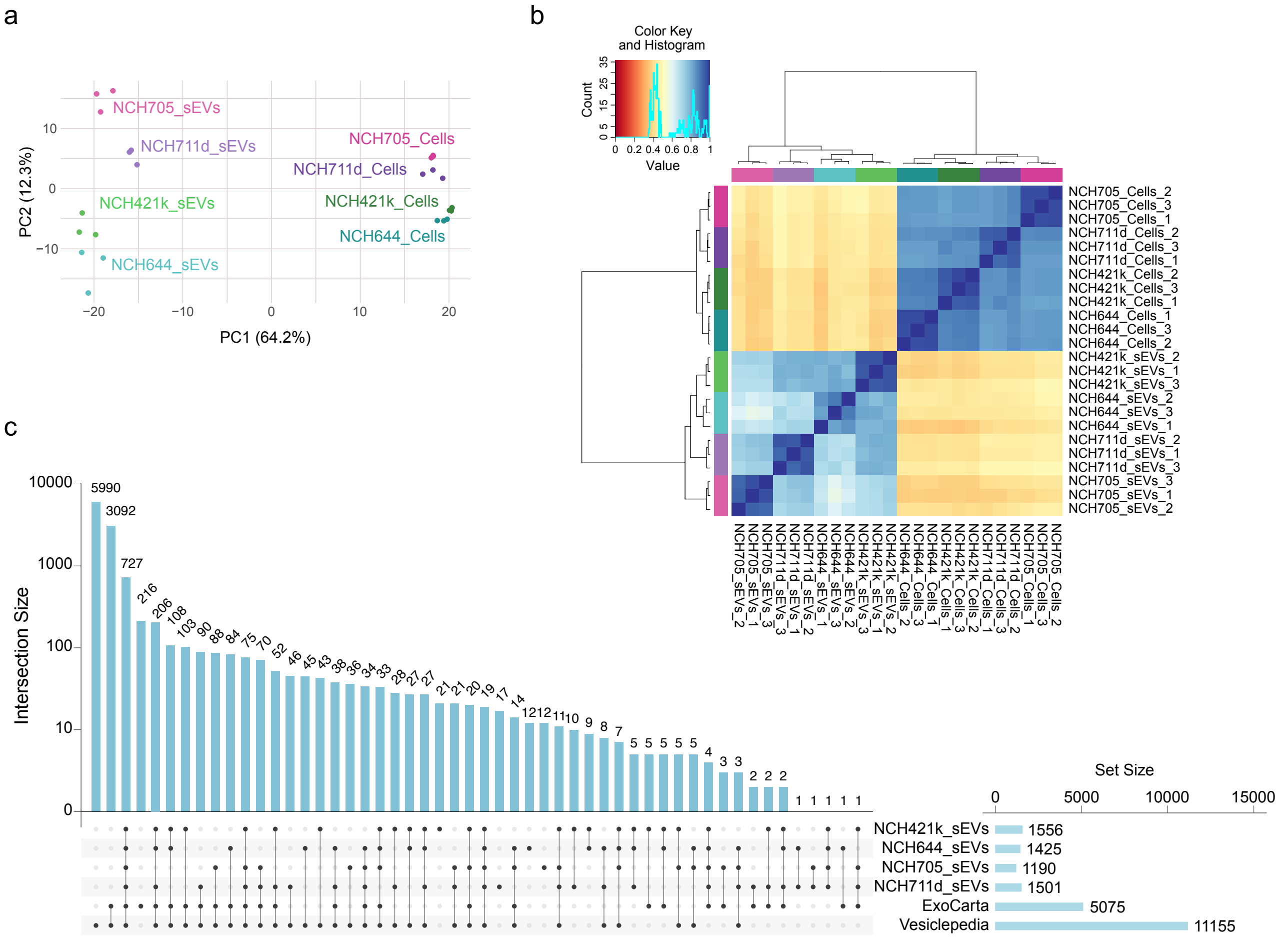


**Supplementary Figure S3.** Proteome profiling of GSC-derived sEVs and their parental cells. (a) Principal component analysis (PCA) of $\log_2$ transformed LFQ intensities. (b) Unsupervised hierarchical clustering of the Pearson correlations for all sEVs/cells samples. (c) Upset plot indicating the number of unique and shared proteins found in GSC-derived sEVs (in all triplicates) and Vesiclepedia/ExoCarta vesicle proteome datasets. The total set size in each data group is shown at the bottom right of the plot. Interconnected circles in the matrix indicate the intersecting proteins.

Supplementary Figure S4

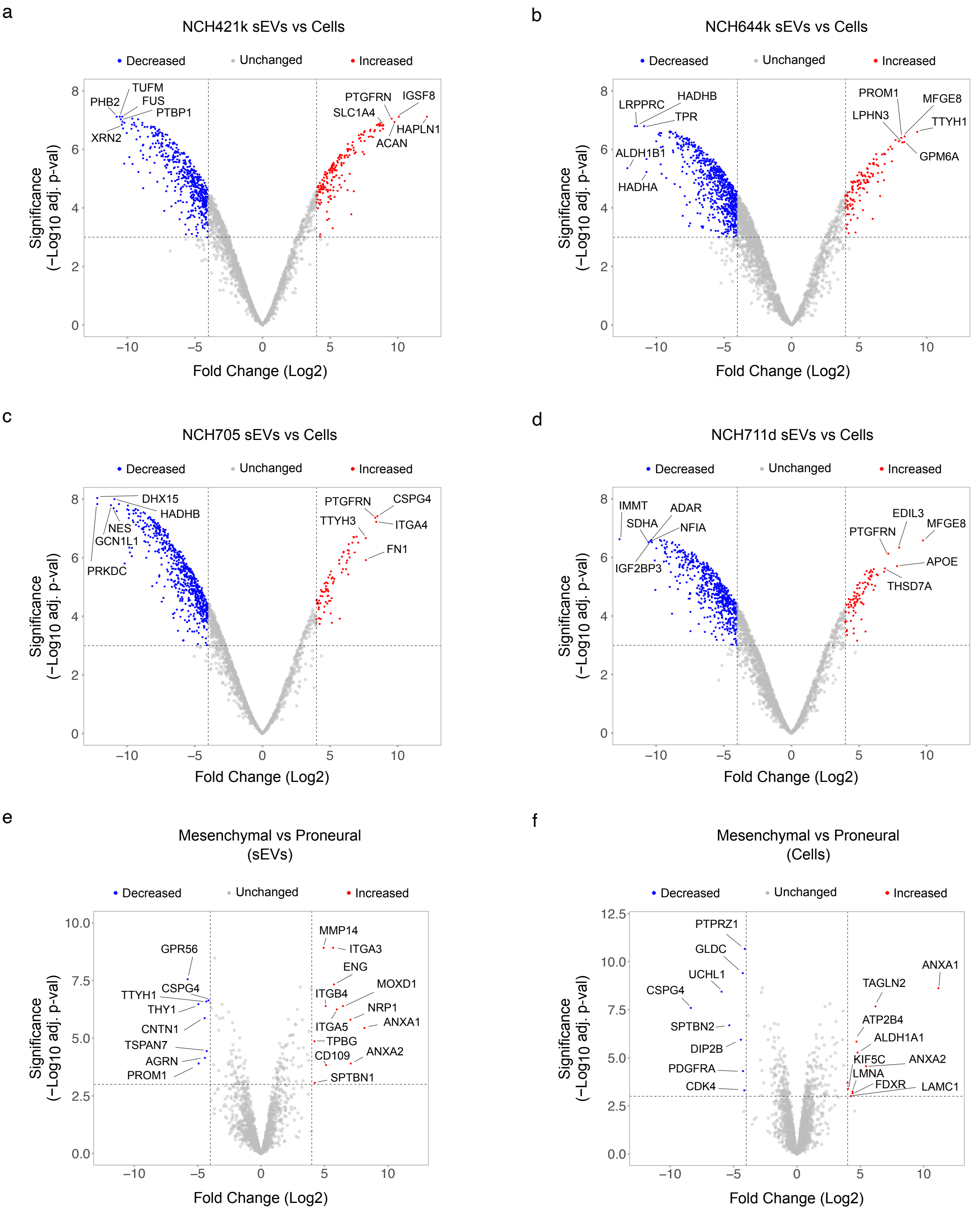


**Supplementary Figure S4.** Volcano plots of quantified proteins in sEVs and whole-cell lysates. Volcano plots show the differentially expressed proteins between sEVs and whole-cell lysates in NCH421k (a), NCH644 (b), NCH705 (c), and NCH711 (d). MES versus PN sEVs and cells comparisons were depicted in (e) and (f), respectively. The horizontal and vertical dashed lines indicate the thresholds of adjusted p-value of 0.001 (-log10 adjusted p-value:3) and fold change of ± 16 ($|Log_2FC|=4$), respectively. Proteins with LFQ or iBAQ values that are completely missing in one condition were not displayed in volcano plots. The protein names indicated in each plot represent the top deregulated proteins in each comparison.

Supplementary Figure S5

a

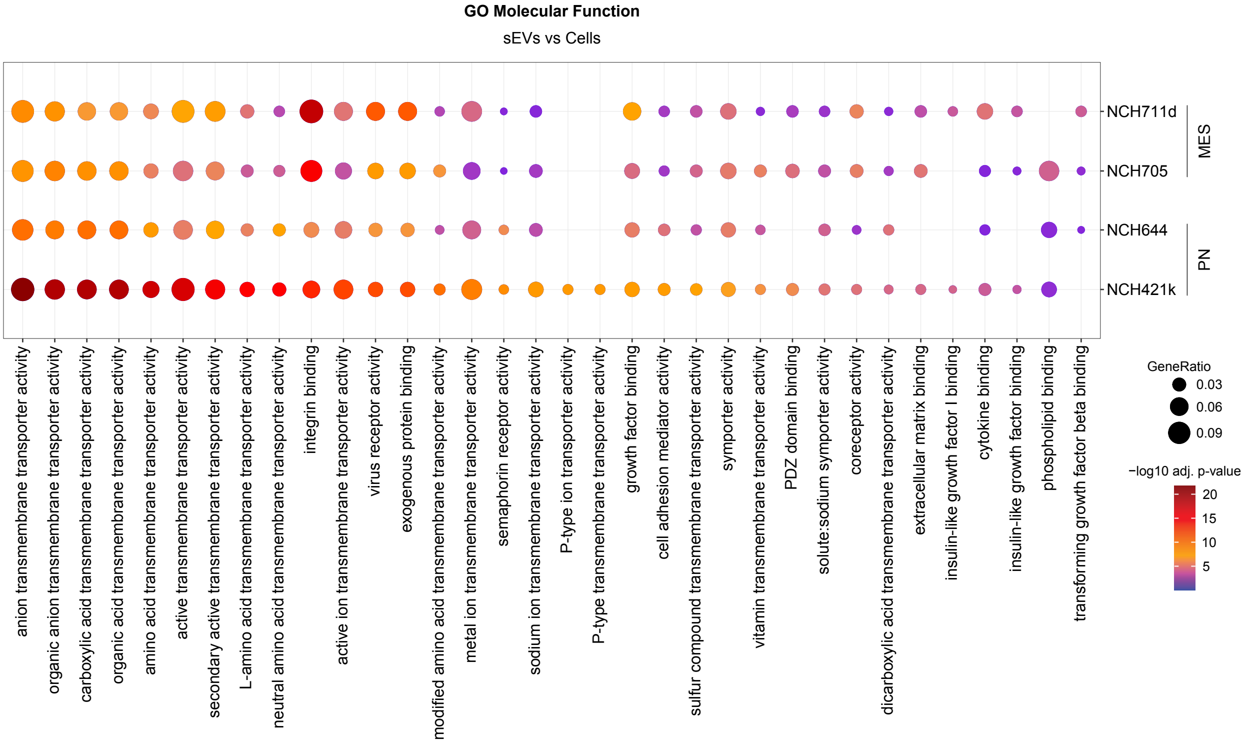


b

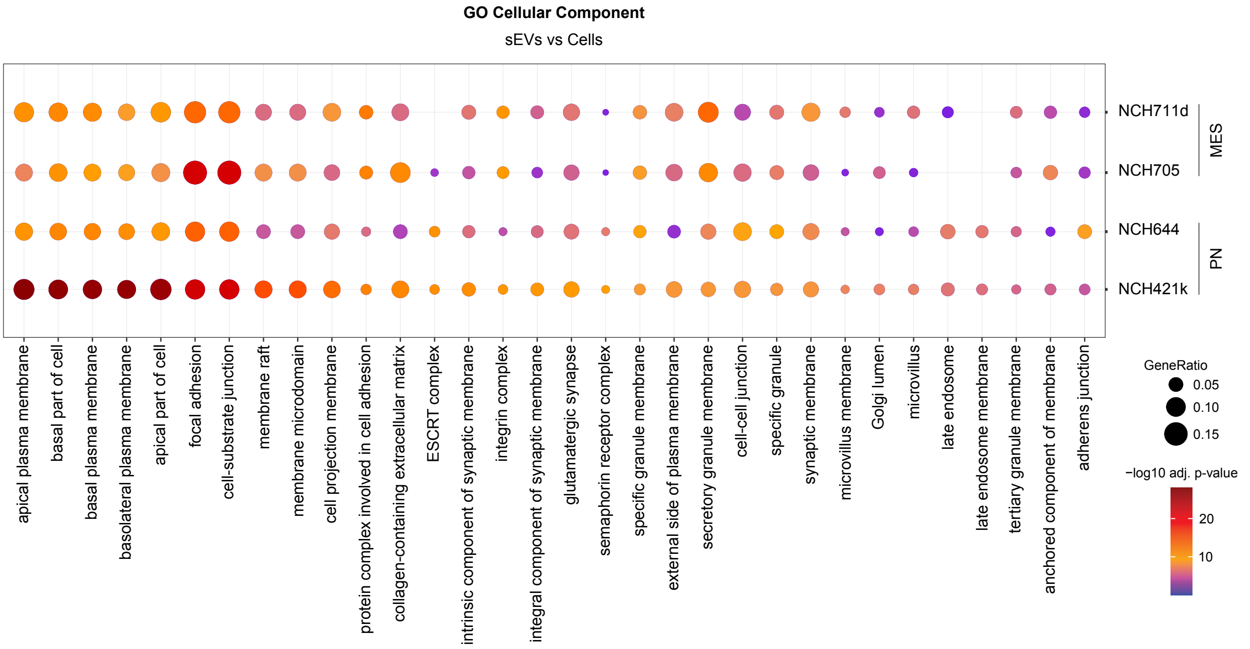


**Supplementary Figure S5.** Gene ontology (GO) enrichment analysis of proteins enriched in sEVs in comparison to their respective cell lines. Dot plots display GO molecular function (a) and cellular component (b) analysis of differentially abundant proteins (sEVs vs cells, adj. pval<0.001 and $log_2FC>4$) in sEVs. The plot was generated by combining top 20 molecular function/cellular component terms of each sEVs versus cells comparisons.

Supplementary Figure S6

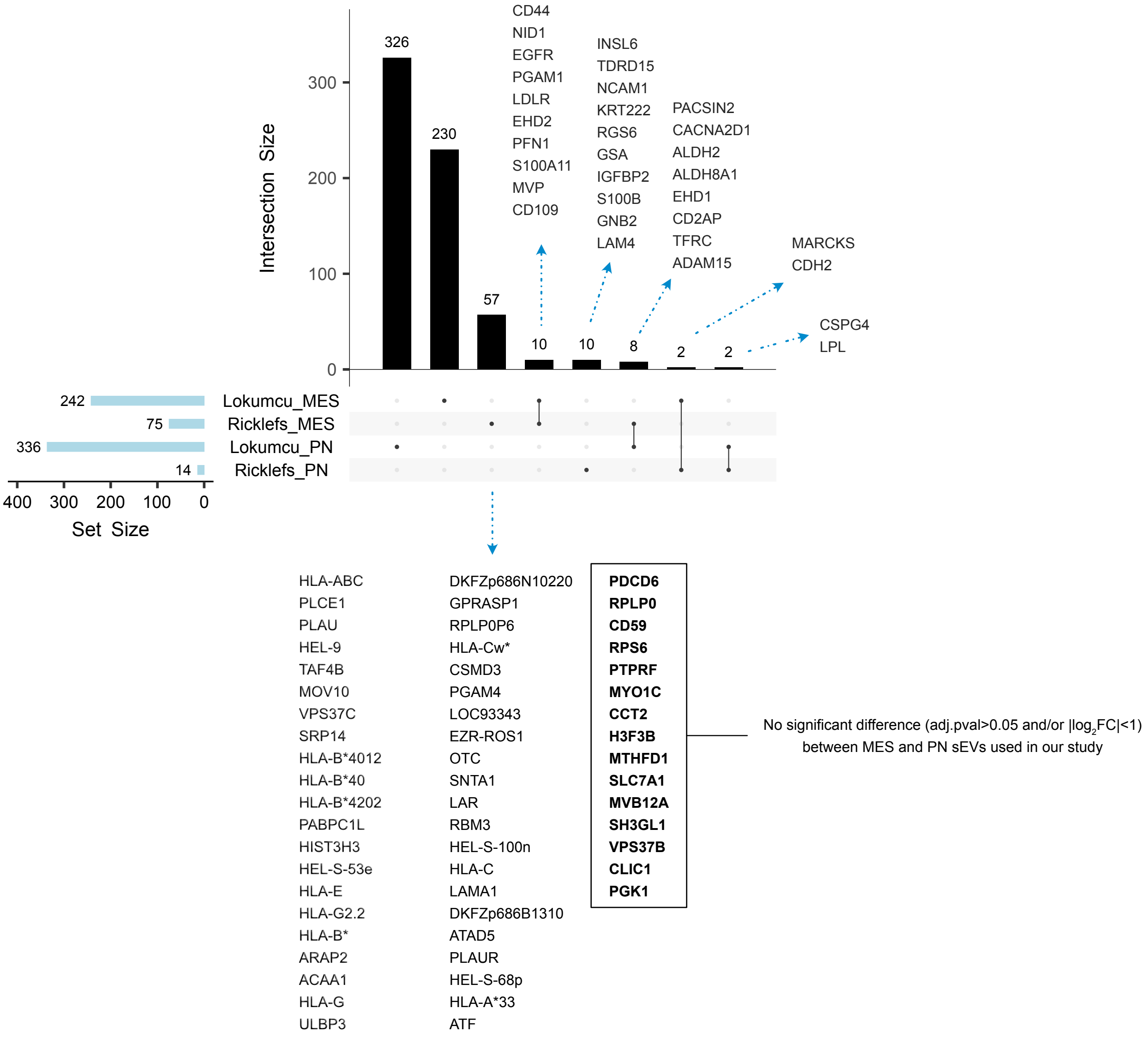


**Supplementary Figure S6.** Upset plot indicating unique and shared proteins found in our and Ricklefs GSC-derived sEVs proteome dataset. The total set size in each data group is shown at the bottom left of the plot. Interconnected circles in the matrix indicate the intersecting proteins. The proteins highlighted in bold are those which have previously been reported by Ricklefs et al. to distinguish mesenchymal (MES) sEVs from proneural (PN) sEVs, but showing no significant difference (adj.pval>0.05 and $|\log_2 FC|<1$) between MES and PN sEVs used in our study. For full list of proteins, please refer to Supplementary file 1.

Supplementary Figure S7

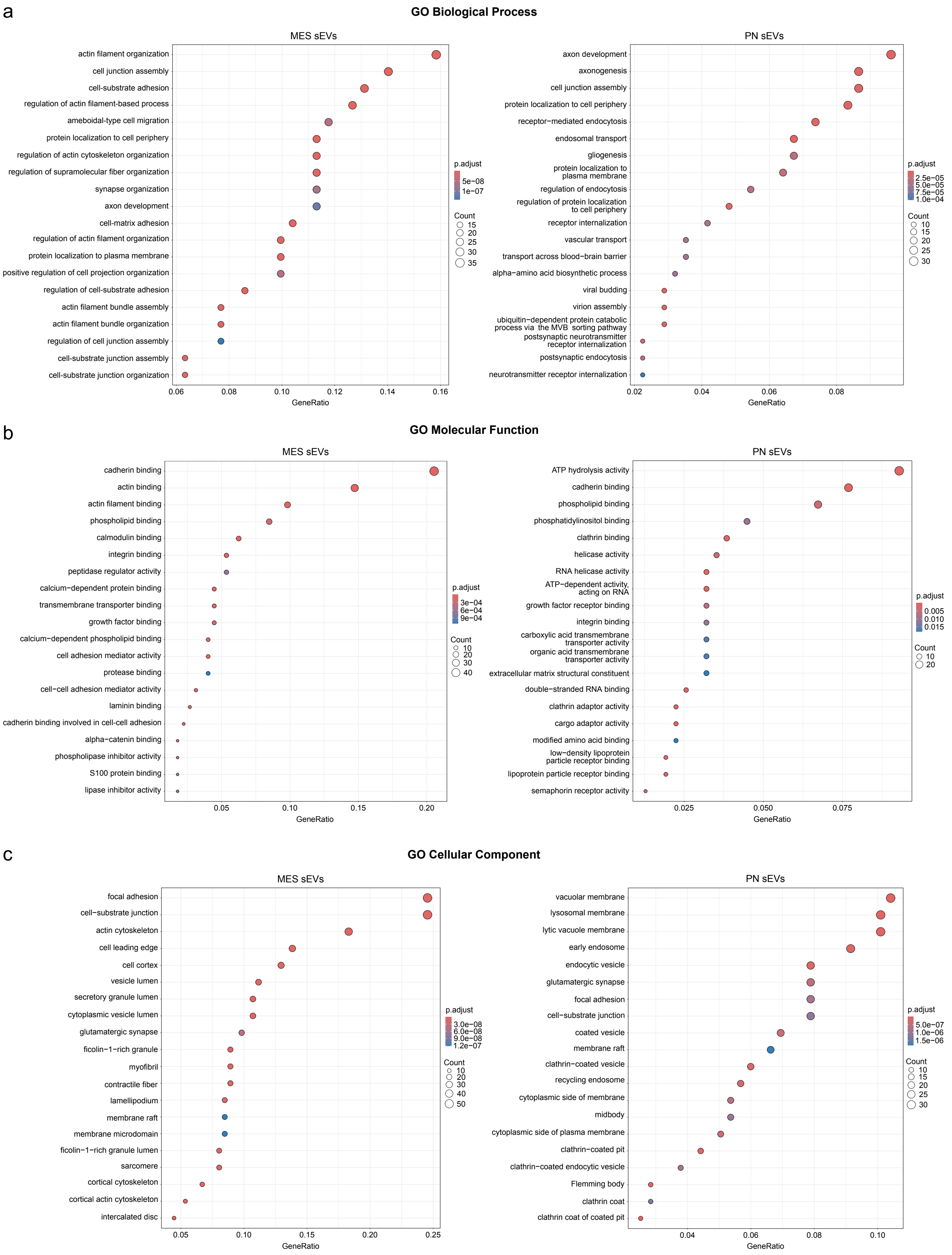


**Supplementary Figure S7.** Gene ontology (GO) enrichment analysis of proteins enriched in MES or PN sEVs. Dot plots display GO biological process (a), molecular function (b), and cellular component (c) analysis of differentially abundant proteins (adj. pval < 0.05 and $|\log_2 FC| > 1$) in MES sEVs (left) or PN sEVs (right). The plots show the top 20 most significant GO terms according to the adjusted p-value (Benjamini-Hochberg correction).

Supplementary Figure S8

a

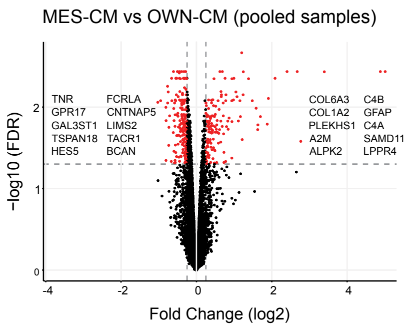


b

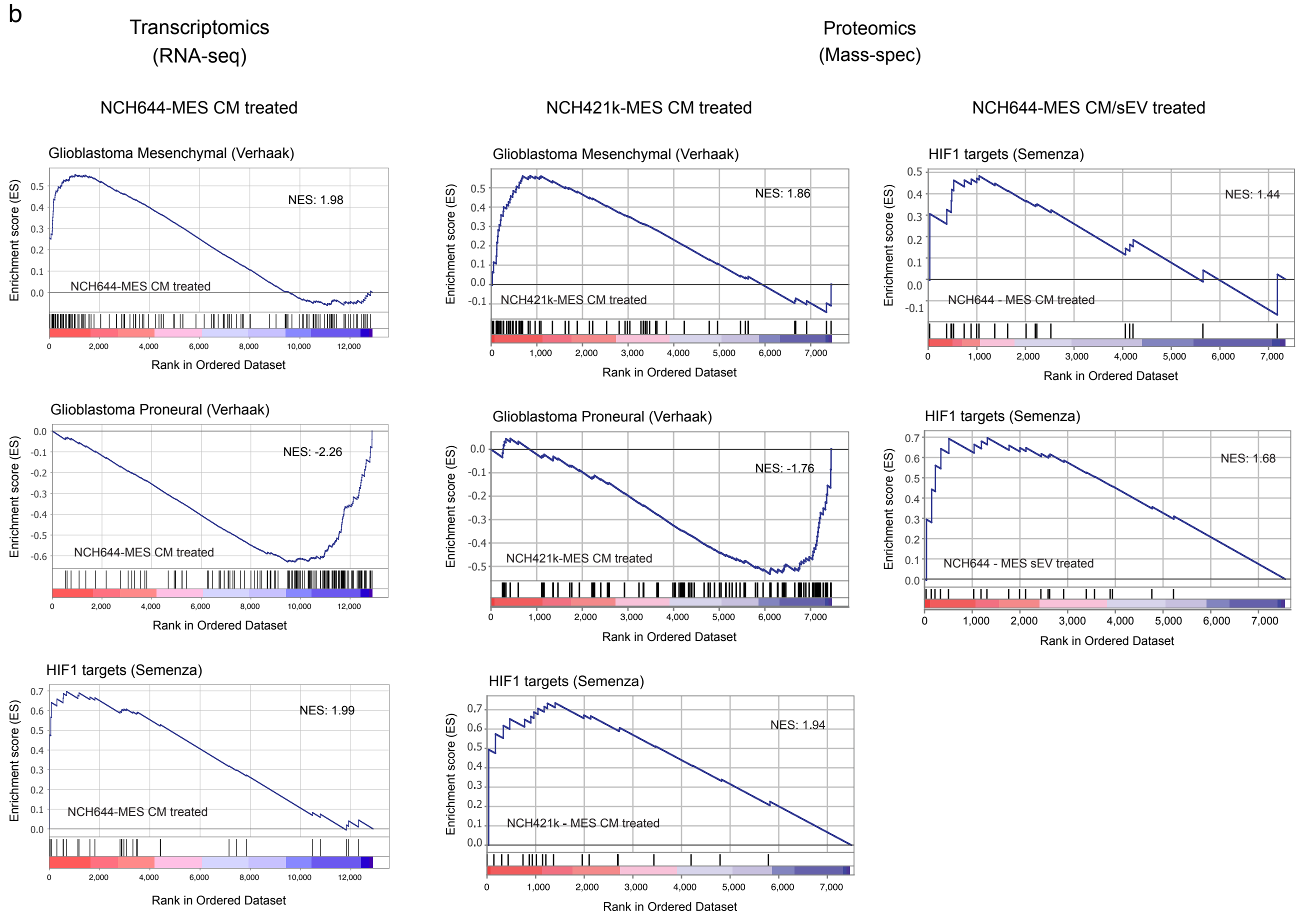


**Supplementary Figure S8.** RNA-sequencing and mass-spectrometry based analysis of PN cells upon the treatment with MES CM/sEVs. (a) Volcano plots demonstrating the differentially expressed genes in PN cells (NCH421k and NCH644 pooled) after the treatment with MES-CM. For simplicity, only 10 up- and down-regulated genes were labelled in the plot. The horizontal and vertical dashed lines indicate the thresholds of FDR of 0.05 and $log_2FC$ of 0.25), respectively. (b) GSEA of transcriptomics data (NCH644 cells treated with MES-CM) and of proteomics data (NCH421k and NCH644 treated with MES-CM and/or sEVs).

a

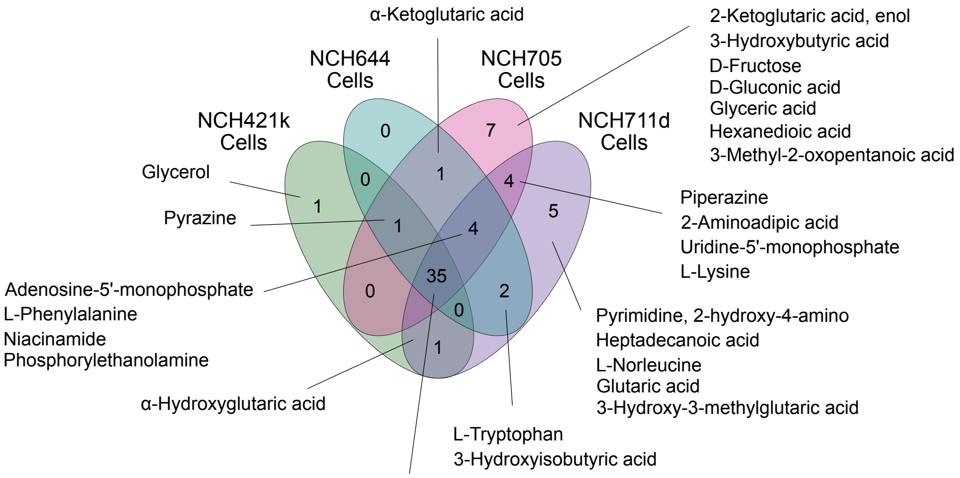


2-Aminomalonic acid
2-Butenedioic acid, (E)-
Erythronic acid
4-Hydroxybutanoic acid
Butanedioic acid
Citric acid
D-Glucose
DL-Phenylalanine
Ethylene glycol

Glycine
L-Alanine
L-Aspartic acid
L-Glutamic acid
L-Isoleucine
L-Leucine
L-Methionine
L-Proline
L-Serine

L-Threonine
L-Valine
Lactic Acid
Malic acid
Myo-Inositol
Pantothenic acid
Glycerol-3-phosphate
Phosphoric acid, monomethyl ester
Pyruvic acid

Glyceric acid-3-phosphate
Propylene glycol
Putrescine
Pyroglutamic acid
Stearic acid
Tyrosine
Uracil
β-Alanine

b

| | Total | Expected | Hits | Metabolites | Raw p-value |
|---|---|---|---|---|---|
| Galactose metabolism | 27 | 0,0708 | 3 | D-Glucose, Glycerol, Myo-Inositol | 1,96E-5 |
| Ascorbate and aldarate metabolism | 8 | 0,0210 | 1 | Myo-Inositol | 0,0208 |
| Glycerolipid metabolism | 16 | 0,0420 | 1 | Glycerol | 0,0414 |
| Starch and sucrose metabolism | 18 | 0,0472 | 1 | D-Glucose | 0,0464 |
| Glutathione metabolism | 28 | 0,0734 | 1 | Pyroglutamic acid | 0,0715 |
| Phosphatidylinositol signaling system | 28 | 0,0734 | 1 | Myo-Inositol | 0,0715 |
| Inositol phosphate metabolism | 30 | 0,0787 | 1 | Myo-Inositol | 0,0765 |

| | Total | Expected | Hits | Metabolites | Raw p-value |
|---|---|---|---|---|---|
| Galactose metabolism | 27 | 0,0531 | 2 | D-Glucose, Myo-Inositol | 8,96E-5 |
| Ascorbate and aldarate metabolism | 8 | 0,0157 | 1 | Myo-Inositol | 0,0157 |
| Starch and sucrose metabolism | 18 | 0,0354 | 1 | D-Glucose | 0,0350 |
| Pyruvate metabolism | 22 | 0,0433 | 1 | Lactic acid | 0,0427 |
| Glycolysis / Gluconeogenesis | 26 | 0,0511 | 1 | Lactic acid | 0,0503 |
| Phosphatidylinositol signaling system | 28 | 0,0551 | 1 | Myo-Inositol | 0,0541 |
| Inositol phosphate metabolism | 30 | 0,0590 | 1 | Myo-Inositol | 0,0579 |

c

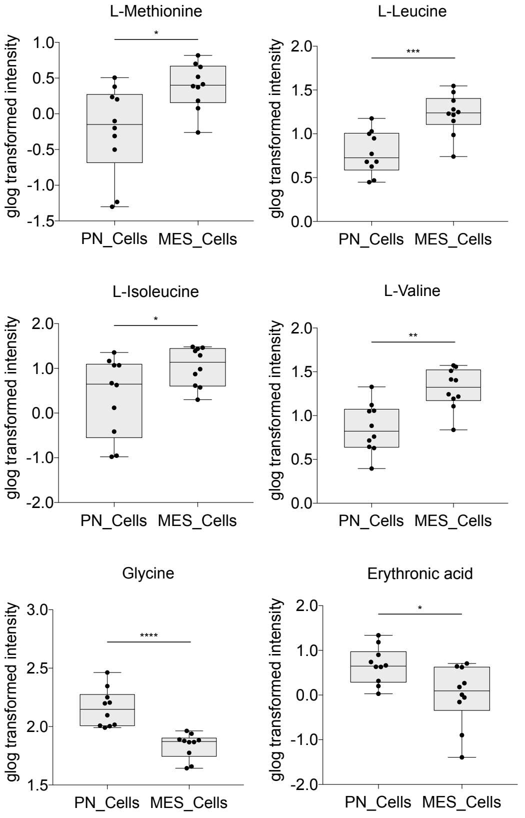


**Supplementary Figure S9.** Metabolites identified in PN/MES GSCs and MSEA results of GSC-derived sEVs. (a) Venn diagram demonstrating the metabolites detected in at least three out of five replicates of a given GSC sample. (b) Metabolite set enrichment analysis (MSEA) results of metabolites identified in PN (top) and MES (bottom) sEVs. (c) Box plots indicating the metabolites that are differentially abundant either in PN or MES GSCs. The sum-normalized intensities of metabolites were generalized logarithm-transformed and statistically compared by unpaired t-test (two-tailed). * p<0.05, ** p<0.01, *** p<0.001, **** p<0.0001.

Supplementary Figure S10

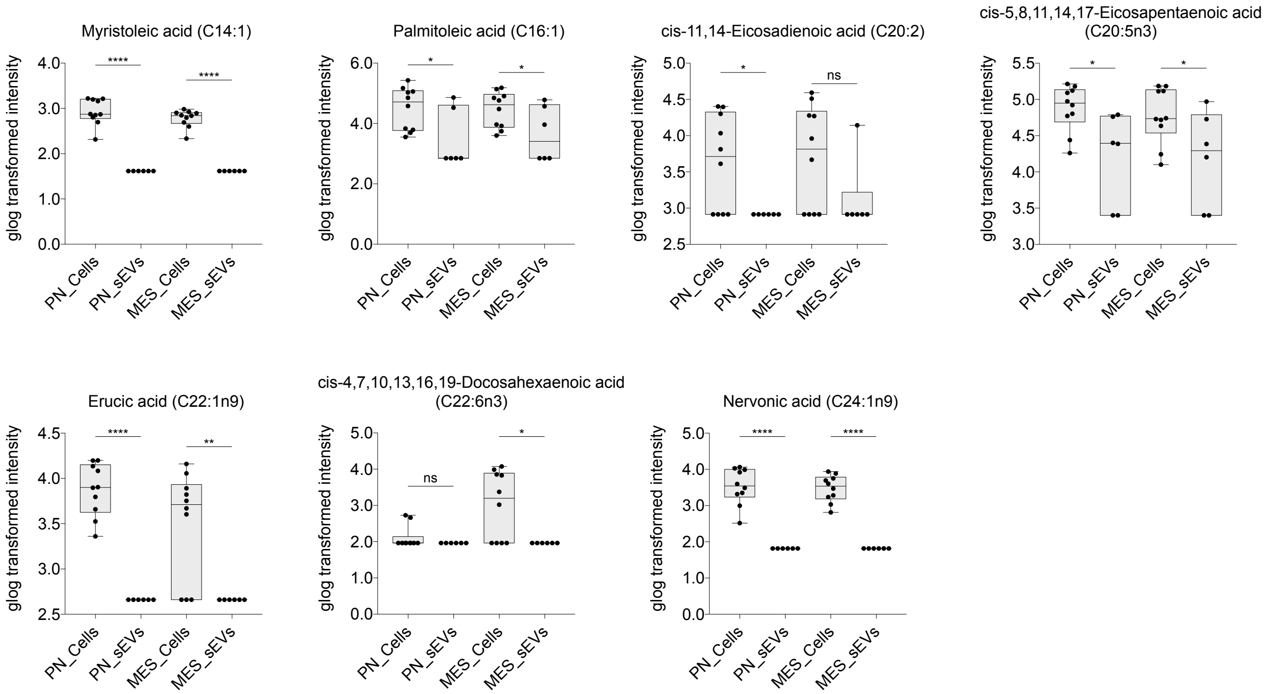


**Supplementary Figure S10.** Box plots displaying the free fatty acids that are significantly low in PN and/or MES sEVs compared with their respective parental cells. The normalized intensities of fatty acid were transformed by generalized logarithm transformation. The p-values for PN cells versus sEVs and MES cells versus sEVs were determined using unpaired t-test (two-tailed). * p<0.05, ** p<0.01, **** p<0.0001, ns: not significant.

Supplementary Figure S11

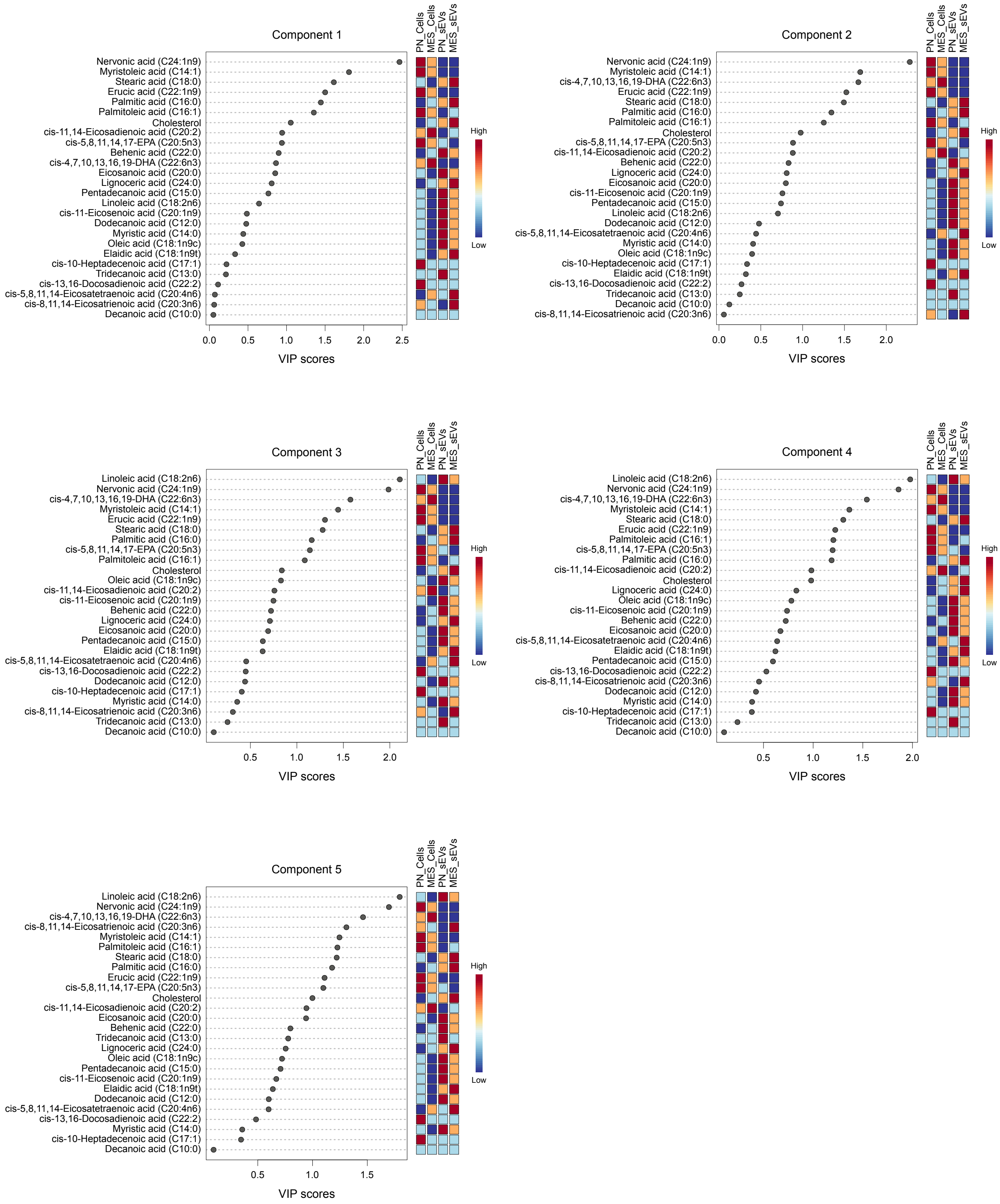


**Supplementary Figure S11.** Variable importance in projection (VIP) score plots showing the important features that differed in PN and MES sEVs/cells. The plots display VIP rank-scores of quantified fatty acids and cholesterol for each component (1-5). The colored boxes indicate the relative abundance (red: high and blue: low) of the corresponding fatty acid in each group.
